# Bioengineering Outlook on Cultivated Meat Production

**DOI:** 10.3390/mi13030402

**Published:** 2022-02-28

**Authors:** Ivana Pajčin, Teodora Knežić, Ivana Savic Azoulay, Vanja Vlajkov, Mila Djisalov, Ljiljana Janjušević, Jovana Grahovac, Ivana Gadjanski

**Affiliations:** 1 Department of Biotechnology and Pharmaceutical Engineering, Faculty of Technology Novi Sad, University of Novi Sad, Bulevar cara Lazara 1, 21000 Novi Sad, Serbia; ivana.pajcin@uns.ac.rs (I.P.); vanja.vlajkov@uns.ac.rs (V.V.); johana@uns.ac.rs (J.G.); 2 Center for Biosystems, BioSense Institute, University of Novi Sad, Dr Zorana Djindjica 1, 21000 Novi Sad, Serbia; teodora.knezic@biosense.rs (T.K.); mila.djisalov@biosense.rs (M.D.); ljiljana.janjusevic@biosense.rs (L.J.); 3 Department of Human Molecular Genetics and Biochemistry, Sackler Faculty of Medicine, Tel Aviv University, Tel Aviv 69978, Israel; savic@tauex.tau.ac.il

**Keywords:** cultured meat, cultivated meat, cell-based meat, cellular agriculture, bioengineering, tissue engineering, bioreactor, sensor, microcarrier, scaffold, medium

## Abstract

Cultured meat (also referred to as cultivated meat or cell-based meat)—CM—is fabricated through the process of cellular agriculture (CA), which entails application of bioengineering, i.e., tissue engineering (TE) principles to the production of food. The main TE principles include usage of cells, grown in a controlled environment provided by bioreactors and cultivation media supplemented with growth factors and other needed nutrients and signaling molecules, and seeded onto the immobilization elements—microcarriers and scaffolds that provide the adhesion surfaces necessary for anchor-dependent cells and offer 3D organization for multiple cell types. Theoretically, many solutions from regenerative medicine and biomedical engineering can be applied in CM-TE, i.e., CA. However, in practice, there are a number of specificities regarding fabrication of a CM product that needs to fulfill not only the majority of functional criteria of muscle and fat TE, but also has to possess the sensory and nutritional qualities of a traditional food component, i.e., the meat it aims to replace. This is the reason that bioengineering aimed at CM production needs to be regarded as a specific scientific discipline of a multidisciplinary nature, integrating principles from biomedical engineering as well as from food manufacturing, design and development, i.e., food engineering. An important requirement is also the need to use as little as possible of animal-derived components in the whole CM bioprocess. In this review, we aim to present the current knowledge on different bioengineering aspects, pertinent to different current scientific disciplines but all relevant for CM engineering, relevant for muscle TE, including different cell sources, bioreactor types, media requirements, bioprocess monitoring and kinetics and their modifications for use in CA, all in view of their potential for efficient CM bioprocess scale-up. We believe such a review will offer a good overview of different bioengineering strategies for CM production and will be useful to a range of interested stakeholders, from students just entering the CA field to experienced researchers looking for the latest innovations in the field.

## 1. Introduction

By 2100, global food systems will need to meet the dietary demands of more than 10 billion [1,2] people who on average will be wealthier than people today and will aspire to the type of food choices currently available only in high-income countries [3]. This food will have to be produced in a sustainable way, contributing to a reduction in climate change effects and addressing other environmental challenges. These requirements are reflected in several of the UN Sustainable Development Goals (SDG), particularly: —zero hunger; SDG3—good health and well-being and SDG13—climate action [4,5,6].

In order to put topics of this review in the context of the current state of the world in the year 2022 and emphasize the need for advances in all the aspects, including the bioengineering-focused ones, that are related to bringing CM closer to real-life use, we remind the readers that the global outbreak of the SARS-CoV-2 zoonotic virus that has been on-going since early 2020 has increased general attention given to zoonotic viral infections [7]. Although COVID-19 is not related to livestock animals, there are other zoonotic viral diseases (e.g., H5N1 [8] and H7N9 avian flu [9]), the risk of which can be associated with livestock and domestic poultry. This highlights a strong need to move away from traditional animal, slaughter-based farming and reduce the number of animals we use in food production, in order to prevent future zoonotic disease outbreaks. Switching to CM or at least complementing the traditional meat and poultry sources by CM products may assist in this prophylactic activity. Equally important are the ethical aspects of animal welfare as well as consumers’ preferences for using primarily slaughter-free food products.

Alternative methods of generating meat products such as production via cellular agriculture (CA), i.e., tissue engineering for food production, have been gaining significant attention as well as massive venture capital funding in the past few decades.

Cultured—cultivated—cell-based meat (CM) as well as cultivated seafood and poultry is regarded as a “hot topic” for investors, with a number of companies such as Aleph Farms, Mosa Meat, Shiok Meats, Upside Foods, etc., gathering large investments for their CM research. Importantly, there is still not a commercial CM product fabricated at a large scale and sold on the global market. The only approved product is a cultured chicken nugget by Eat Just, branded as GOOD meat, approved in December 2020 by the Government of Singapore, with additional GOOD meat chicken product formats being approved in 2021. However, even these products are currently being sold only as a limited series in Singapore. Up to now, the only contract manufacturing facility for CM production in the world is Esco Aster that in September 2021 received regulatory approval in Singapore to produce CM for commercial sale.

This short recapitulation of the current CM industry environment indicates several important points: (1) a large portion of CM research is being conducted in privately funded companies, making any discovery a proprietary intellectual property, not available for re-use and improvement in the general scientific community. There are attempts to rectify this, primarily by non-profit foundations providing grants for open science research projects, such as the Good Food Institute and New Harvest; however, the main part of CM inventions still stays within the proprietary perimeters of the companies; (2) despite significant breakthroughs and large cost reductions in the CM bioprocess versus initial costs for the first CM burger made in 2013 (for which the costs were amassing EUR 330,000 for 1 patty), we are still far from large-scale facilities for CM production. The authors of this review believe there is a clear correlation between these two insights—keeping new CM findings only as proprietary information and not advancing significantly towards the scaling up of CM bioprocess. This review is one attempt to assist research groups interested in starting and/or continuing CM research by presenting the current knowledge of several main aspects of CM bioengineering, i.e., tissue engineering for CM production, such as cell sources and components for adherent cells’ immobilization such as microcarriers and scaffolds, bioreactor types, cultivation media requirements, options for bioprocess monitoring and modeling of the bioprocess kinetics. We also provide several directions in which we believe CM research needs to focus and invite all the readers to contribute with their open science approach towards making these a reality.

## 2. Major Cell Types with Potential for Use in Cultivated Meat (CM) Bioprocess

Meat is a very complex structure, containing several types of different tissues combined into a well-organized architecture including muscle, adipose, skeletal and vascular components [10,11]. CA and CM fabrication is an attempt to generate meat structures in in vitro conditions. Achieving CM with the same characteristics as “native meat” would mean proliferating and differentiating stem/progenitor cells to mature tissues that constitute meat, by keeping their biological variety and nutritional values.

### 2.1. Pluripotent Cells

Firstly, by addressing the issue of division and differentiation as part of cellular self-renewal ability, scientists started employing pluripotent embryonic stem cells (ESCs) for their potential to differentiate into almost any cell type, while still keeping the ESC colony fully active [12]. ESCs are explanted to form a cell culture implantationally—delayed blastocysts, specifically from the inner cell mass [13]. Their infinite division is due to the unique identity of ESCs, a rich network of transcription and epigenetic factors that program their pluripotency [14,15]. However, ESCs’ pluripotency decreases in their offspring cells to a major extent through a process of forming more specialized multipotent stem cells [16]. Mesenchymal stem cells—MSCs—for instance keep their quiescence and the ability to potentially activate [17,18] and further divide. The latter gives hope that MSCs can be used for further replication, proliferation and full mature tissue formation in the process of CM [19,20]. In vitro culturing of pluripotent stem cells such as ESCs is established, but still represents a very “dirty” process in which the cell-specific differentiation is mediated by selected components of the cell media. Moreover, recent efforts to generate a stable culture of bovine embryonic stem cells delivered from blastocyst-stage embryos were successful [21,22].

Similar to ESCs, induced pluripotent stem cells (iPSCs) keep their ability of almost limitless self-renewal, including asymmetrical division and differentiation [23,24,25,26]. iPSCs are created in a dish from ESCs or somatic/adult stem cells extracted by tissue biopsy (skin, bone or blood cells) and are subsequently subjected to transcriptional modification or reverse reprogramming (respectively) to an embryonic-like pluripotent state [23,24]. Although the cell reprogramming processes in both iPSC and ESC require transcriptional modification and chromatin remodeling, it still remains elusive why only the iPSC differentiation is highly inefficient, giving a variety of low-yield cell types [26,27,28]. As such, iPSC are intensively studied in terms of animal sources (bovine and porcine cells are mostly characterized, while avian cell culturing remains elusive [29]), media optimization for the high yield requirements of the CM industry and new protocols to establish different approaches of IPSC cultivation for CM [21]. For instance, one approach describes culturing stem cells with specialized diets of growth factors and small-molecule inhibitors to cultivate cells from the pluripotent state toward myogenic offspring cells [30]. Another approach shows conditional use of different transcription factors to evoke direct and efficient differentiation [31,32]. Alternatively, some authors suggest significant yield rise by the maintenance and differentiation of pluripotent stem cells in serum-free [33], animal-component-free cell culture medium [34] and a carrier-free suspension environment [35].

### 2.2. Multipotent Cells

Adult stem cells—ASCs—also called tissue-specific or somatic stem cells, are multipotent, undifferentiated progenitor cells located in specific animal organs and tissues. They are potentially one of the best sources of starting cells to be used in the CM production. Three major stem cell types that have been studied for their ability to initiate CM production are mesenchymal stem cells (MSCs), fibro/adipogenic progenitors (FAPs) and satellite cells (i.e., myosatellite cells or muscle precursor cells)—SCs. When combined, these cell types have the ability to differentiate into all of the cells present in meat [36]. However, since SCs have proven to be the most promising, in the last several decades they have been used for CM experimental production, together with myoblasts [37,38]. The world’s first CM burger prototype was produced by amplifying the bovine SC/myoblast progeny [39], and muscle tissues produced by SC culture are considered a great source of protein [38].

When considering MSCs, it is important to emphasize they can be sourced directly from various tissues, such as the stromal–vascular fraction of skeletal muscle, bone marrow, umbilical cord, and adipose tissue [36,40,41], or by differentiating the ESCs/iPSCs [42] and further modifying them into mature myocytes, adipocytes, and fibroblasts [43,44]. Reprogramming of MSCs by media supplementation and specific pathway inhibitors can help overcome their poorer differentiation abilities and propagate new cell-type biogenesis and proliferation [45,46,47,48].

Another notable multipotent cell type considered for use in CM production is adipose tissue-derived ASCs. Obtaining these cells from certain animals’ subcutaneous fat is a non-invasive procedure, after which they can differentiate into adipogenic cells or transdifferentiate into osteogenic, myogenic or chondrogenic cells ex vivo [49]. However, it is also very important to point out that adipose tissue-derived ASCs have a tendency for malignant transformation in long-term cultures [50].

Skeletal muscle satellite cells (SCs) are myogenic cells located beneath the basal membrane of terminally differentiated skeletal muscle fibers (myofibers) and their primary role is in muscle development. They are in a quiescent state and act as a reserve population of cells. From this reserve pool, SCs can increase their own numbers or differentiate into myocytes, which, in turn, form myotubes that organize themselves into non-proliferative myofibers. After the activation of dormant SCs, they transform to actively proliferating cells called myoblasts. Isolation and in vitro maintenance of SCs from livestock, i.e., agriculturally important species such as cattle [51,52,53,54,55], chickens [56,57,58], sheep [59], pigs [57,60,61] and rabbits [54] have been well optimized. Additionally, for potential application in CM production, research has also been conducted with fish SCs [36,62,63,64]. On the other hand, protocols for obtaining skeletal myocytes [46,47], and SCs [48] from mouse and human PSCs have also been well optimized.

Although ASCs can be easily and affordably obtained, and they are still capable to differentiate into the mature cells that need to be present in meat, there are limits to their proliferation capacity and maintenance in vitro [36,65]. Further improving the proliferative capacity of ASCs represents one of the different approaches to achieve scaling up of the selected cell source, which is a very important consideration in developing the CM production bioprocess. Achieving scale-up of SCs can be realized by keeping them in a non-differentiated, proliferative state, where this effect can be prolonged by in vitro inhibition of the p38 mitogen-activated protein kinase (MAPK) cell signaling pathway [29,53].

In addition to using animal single myogenic cell lines in CM production, it is necessary to co-culture multiple cell types in order to develop functional 3D skeletal muscle. However, it needs to be emphasized that this approach is very challenging because each cell type has its own specific cultivation needs. Additionally, there are a number of methods for the co-cultivation of cells. For example, to produce a fully formed steak, Aleph Farms co-cultivated three different cell types using a plant-based 3D porous scaffold: SCs, endothelial cells and extracellular matrix (ECM)-secreting cells such as fibroblasts [66]. Shima et al. co-cultured C2C12 myoblasts with 3T3 fibroblasts using cell fiber technology, i.e., cells were 3D co-cultured by encapsulation in the cell-laden hydrogel microfibers [67]. Jo et al. formed a microtissue composed of microfiber-based adipose tissue on a polydimethylsiloxane (PDMS) substrate with myoblast-laden collagen solution covering on top [68]. In order to produce healthier and thicker cultured food, animal cells can be co-cultivated with algae for 3D tissue fabrication. Thus, Haraguchi and Shimizu co-cultivated C2C12 cells and photosynthetic autotrophic microalgae *Chlorella vulgaris* (*C. vulgaris*) [69].

Although the most common method for obtaining cells required for CM production is minimally invasive sampling from a live animal, well-established continuous cell lines (i.e., immortalized cells) could be a useful alternative source, particularly when considering industrial-scale production.

While immortalized cell lines of different livestock species are not yet available, there is a great need for this approach due to its cost- and time-effectiveness, as well as eliminating the harming of animals. However, one of the main limitations of the use of these cells is that they are not always representative of the primary culture due to potential spontaneous mutations during long-term cultivation [20,50].

In order for immortalized cells to be used in CM production, there are conditions that must be met. Firstly, the cell lines must be safe to use, then they must be adequate for large-scale production, and finally they need to be desirable to consumers [70]. Additionally, for each potentially applicable cell type, it is necessary to establish cell isolation protocols, culturing conditions and medium formulations.

**Figure 1:**
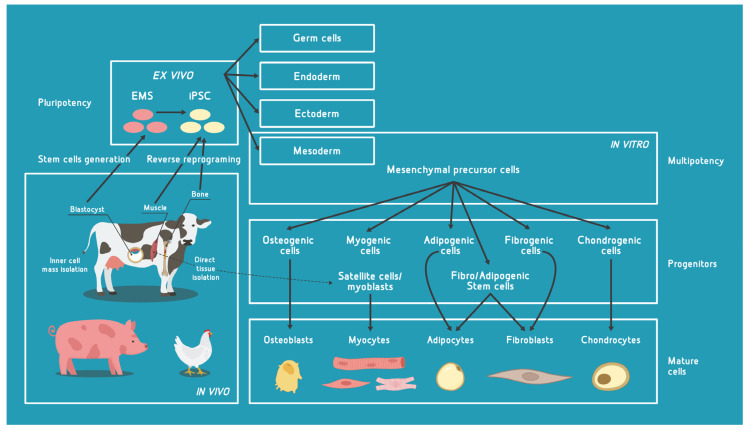
Major cell types of interest for CM bioprocess.

In Figure 1, we give an overview of the major cell types currently used in relation to CM and their sources.

### 2.3. Cell Immobilization

#### 2.3.1. Microcarriers

The bioprocess of CM production can generally be divided into four phases [71]. The first phase (I) involves cell isolation and initial proliferation; the second phase (II) represents cell expansion on a large scale (i.e., in expansion bioreactors); the third phase (III) is cell differentiation or tissue formation in other types of bioreactors; and finally, the fourth phase (IV) involves processing into a final food product [71].

Micro-scale cultures of microcarriers (MCs) made of synthetic or natural polymers have been developed for efficient proliferation of anchor-dependent cell sources necessary for the CM production bioprocess on a larger scale. Therefore, considering their high surface-to-volume ratio, MCs play a major role in the second phase of the CM production bioprocess because they allow an increase in the total biomass of undifferentiated cells [71,72,73].

A variety of commercially available MCs are designed for cells used in the biomedical field, such as iPSCs [74], ESCs [75,76] and human MSCs (hMSCs) [77,78], and a significantly smaller number of MCs are designed for use in CM production [71]. As described in detail in Section 2.1 and Section 2.2, notable cell types for CM production are muscle SCs/skeletal myoblasts [37], MSCs, FAPs, ESCs and iPSCs, obtained from relevant species such as cattle [54], chickens, pigs, sheep [36] and rabbits [54].

Some of the advantages of MCs are the simplicity of their production in large quantities, easy implementation in various bioreactors [73,79] and efficiency in the scale-up proliferation of adherent cells. On the other hand, MCs also have several limitations, most depending on their cost and whether they are edible or non-edible.

Edible MCs for use in CM production can be incorporated into the final food product without the need for a dissociation or separation step [72,73] and they could potentially have a positive impact on the texture, nutritional and/or organoleptic properties of the product [36]. They can be composed of natural or synthetic polymers, such as polyglycolic acid (PGA) and polyethylene glycol (PEG), that are inert. Natural polymers from which MCs are made include various polysaccharides of plant origin (e.g., starch), animal origin (e.g., chitosan) and algae origin such as alginate [80]. Additionally, they include lipids such as paraffin and shellac [81] and polypeptides such as zein [82], collagen, gelatin, pectin and cardosin A [83,84]. These types of polymers and materials derived from them are easily obtained and can be biocompatible [85] as well as biodegradable [73,86,87]. Although few in number, there are companies that make edible MCs for use in CM bioprocess, such as Matrix Meats [88] and Tantti Laboratory [71].

On the other hand, the use of non-edible MCs, such as those containing poly(lactic-co-glycolic acid) (PLGA) in the CM production bioprocess, can introduce food safety concerns and require a dissociation, degradation or separation step involving multiple treatments such as mechanical/thermal/chemical, which is complicated, less desirable, costly and time-consuming. Additionally, this step can reduce the total yield of cellular biomass due to inefficient detachment or cell death [72].

Although still on a smaller scale, a number of alternatives to MC cultures based on organoids [89], spheroids [90,91,92] or single-cell suspension culture [93] are being considered [71].

Thermo-responsive MCs were also investigated considering the feasibility of thermally induced cell detachment after expansion. Yuan et al. [94] proposed thermally responsive poly(*N*-isopropylacrylamide) grafted MCs (PNIPAM-MCs) for expansion of hMSC, which was carried out in spinner flasks and a PBS-VW (Vertical Wheel) bioreactor, while subsequent cell detachment after expansion was performed at room temperature (23 °C). This method of cell detachment from MCs provides the possibility to avoid trypsinization, contributing to the reduction in total bioprocess cost considering simplified downstream processing without additional chemicals included, as well as reduced energy requirements, since the cell detachment is performed at room temperature.

The appropriate choice of MCs should also consider the material they were made of from the point of view of cells’ affinity to bind to MC surface, as well as the type and strength of the formed physico-chemical bonds in order to provide cell retention during cultivation and simplified cell detachment in the post-harvest processing, if necessary.

#### 2.3.2. Scaffolds

Scaffolds are 3D structures necessary for the complete in vitro bioprocess of tissue formation. They consist of a porous material, provide mechanical support and an integrated network—they mimic the in vivo environment of living cells, i.e., ECM, in order for adherent cells to bind and differentiate into the necessary cell types. Scaffolding material can enable the potential vascularization and spatial heterogeneity in the final product, which would bring the texture and structure of CM closer to conventional meat [20,28,95]. The porous structure of the scaffold helps maintain metabolic functions of the cells and prevent necrosis by allowing the continuous perfusion of culture medium, permitting efficient nutrient and oxygen flow, as well as waste product removal [73].

For the application of scaffolds in CA and CM production, there are requirements that must be paid attention. Scaffolds should be biocompatible, biodegradable/edible [29,96], as well as have appropriate mechanical properties such as material strength, thickness, stiffness, pore size, specific texture—scaffold architecture [97,98,99]. The values of the mentioned properties that the scaffold should contain depend on the desired final product. For example, if the desired final product is meat, which consists of both muscle and adipose tissue [100], the scaffold needs to have appropriate stiffness in order to be suitable for both tissues (skeletal muscle tissue need much more stiff and rigid scaffolds than adipose tissue) [97,98]. Additionally, scaffolds should possess certain nutritional values, thermal stability, be non-allergenic and non-toxic, and help organoleptic properties (i.e., pleasant taste) [101,102]. Last but not least, for a better consumer experience, the familiar look and texture of conventional meat is required [103,104].

As for their composition, scaffolds can be made from synthetic polymers or natural edible polymers. The latter can be animal-derived [29], plant-derived [102,105,106], fungi-derived, algae-derived [107] or composites of different polymers [108].

When it comes to natural polymers, the most often still used for CM production are those from animal sources, which are expensive, non-sustainable and have a large environmental impact. Animal-derived scaffolds include primarily gelatin [54] and collagen scaffolds, which are considered to be the gold standard [19,29,39,109]. Large quantities of livestock are necessary as a source of these biopolymers, as they lack self-replicating capabilities [29]. Fibrin is also used to make scaffolds, primarily in the field of tissue engineering (TE) where it has been shown to optimize the vascularization of bio-artificial muscles (BAMs) [95,110,111,112]. Another polymer that is important to mention is hyaluronic acid (HA), where due to the unsustainability of the animal-derived scaffold, alternative sources of HA using endotoxin-free microorganisms (MOs) have been identified [113]. The scaffolds that are also used in the TE field (i.e., bone TE) and have the potential for wider application are silk scaffolds [114]. There is also great interest in keratin scaffolds [115]. Finally, another animal-derived, as well as fungi-derived, biopolymer is chitosan [95,107,116,117]. Apart from this, pullulan is also a biopolymer of fungal origin that has the potential for application in CM production [118].

On the other hand, there is great research interest in plant protein-based scaffolds because they are widely available, affordable, biocompatible, and have high nutritional value [119,120] and a positive impact on environment conservation and animal welfare. A disadvantage of using plant-derived scaffolds is the lack of mechanical properties, which may be resolved either with different physical, chemical, or enzymatic crosslinking processes [95,121] or mixing with materials that have high mechanical properties [121]. The most common plant-derived proteins used to produce scaffolds are zein and soy proteins. The application of edible, highly biocompatible textured soy protein as a CM scaffolding material has been investigated by Ben-Arye et al., which has resulted in complete 3D-engineered bovine muscle tissue [102]. Research conducted by Jiang et al. [122] and Qu et al. [123] has shown that zein scaffolds can provide adequate binding, proliferation and differentiation of specific cells, which gives zein potential for application in CM production [95]. Plant-derived polysaccharides with potential use in CM production include starch, cellulose [124,125] and pectin [126]. In addition to plant polysaccharides, algae-derived polysaccharides are also important. An example is alginate, which Apsite et al. used in scaffolding for the 4D biofabrication of skeletal muscle microtissues [127]. However, special attention should be paid because alginate products can pose a risk of allergies.

Additionally, natural biomaterials for potential scaffold production include composite matrices that can be microbial-derived and plant-derived, such as fungal mycelia [128], lignins, decellularized seaweed cellulose scaffolds [129] and plant tissues (e.g. leaves) scaffolds [130]. For example, Modulevsky et al. have shown that decellularized apple hypanthium can be used as a 3D cell culture substrate [131]. In addition, decellularized jackfruit and spinach leaves, as well as marine macroalgae species *Ulva* sp. and *Cladophora* sp. [129], have been explored as potential scaffolds in TE, so they could possibly be used in CM production as well.

In addition to natural ones, synthetic polymer scaffolds can also be considered for potential use in CM production. The scaffolding material must be safe for human consumption—edible—or biodegradable without making any toxic by-products. The synthetic polymers most commonly used in the TE field are polystyrene, PGA, polylactic acid (PLA) and PLGA copolymer [132]. The advantages of synthetic polymer scaffolds include consistent supply and batch-to-batch reproducibility [133], while, on the other hand, their use can be limited by reduced biocompatibility, production costs and the necessity for surface functionalization [95]. To improve their biocompatibility, these scaffolds can be combined with natural polymers such as isolated proteins or peptides.

Considering the requirements for scaffolds used in muscle TE [134], approximation could be made when it comes to scaffolds used in CM production, which should support muscle cell maturation, as well as meet the following conditions: preferably be edible, allow vascularization and innervation and facilitate directional alignment in order to achieve muscle structure similar to the animal meat. Vascularization in the 3D tissue constructs is one of the major constraints, since proliferating cells themselves represent a barrier for mass transfer, especially when it comes to nutrient and oxygen delivery [135].

## 3. General Aspects of Bioreactors for Proliferation and Differentiation in CM Bioprocess

### 3.1. Two-Dimensional vs. Three-Dimensional Cell Culture for Cultivated Meat Production

The most important disadvantages of 2D planar cell culture include time-consuming feeding and passaging, as well as inconsistency in bioprocess outcome in terms of yield and cell quality due to poor bioprocess control resulting in non-homogenous culture conditions and culture-to-culture variability [136]. Some additional disadvantages of monolayer culturing of animal cells compared to other expansion/differentiation culture systems include limited scale-up capability, dependence on incubators to control temperature, oxygen (O_2_) and carbon dioxide (CO_2_) concentration, high dependence on manual operators considering that manual and time-consuming medium exchange is required to prevent inhibiting effects of metabolic by-products, increasing risk of contamination and total production cost, where the necessity to perform the bioprocess in cleanroom facilities also has a significant contribution [137]. All of the aforementioned disadvantages point out the unsuitability of planar culture for large-scale production of CM. Hence, there is a necessity to develop large-scale and controlled bioprocess solutions with automated bioprocess workflow not only in terms of CM production, but also for expansion of cell culture for TE in regenerative medicine [137,138].

The requirements to increase scale in TE, hence in the production of CM as well, resulted in a shift from 2D planar culture in T-flasks to 3D cell culture, where the application of bioreactors is required to ensure a favorable environment for cell growth and maturation. The emergence of the novel variables, including closer interactions between the cells and the extracellular matrix, in 3D cell culture results in different effects to cell survival, proliferation, differentiation and migration [139].

The lab-scale attempts to provide a 3D environment for animal cell culture have included application of spinner flasks, which provide similar conditions for cultured cells as large-scale industrial bioreactors with internal mixing [140]. The inability to perform precise control of O_2_/CO_2_ supply, as well as pH value control, makes them suitable only for limited bioprocess modeling purposes at the lab scale. However, the bioprocess modeling and optimization are crucial parts of preparing for the scale-up, which makes spinner flasks very successful small-scale model systems, particularly in terms of defining mass and energy transfer models.

Submerged conditions, i.e., cultivation of cells in the liquid medium, especially with the addition of MCs, require adequate mass and energy transfer in order to provide uniform conditions for each cell, mostly in terms of nutrient availability, as well as in terms of temperature and pH value regulation [138]. Therefore, it is important to define mass and energy balances, as well as adequate models describing mass and energy transfer [141]. Stirring and agitation rate and their technical feasibility are the factors with major significance for mass and energy transfer. Therefore, their profound understanding and optimization is a pre-requirement for preserving cell viability and spurring cell proliferation, simultaneously minimizing bioprocess energy input and hence overall bioprocess cost, which becomes even more important at a larger scale with high energy demands for uniform mixing and aeration of the cultivation broth. Considering the current high costs of CM production, mostly due to expensive culture media [142], bioprocess optimization in terms of all aforementioned bioprocess parameters should be performed on a smaller scale using a lower volume of the cultivation medium, and it is usually performed in spinner flasks. Adequate experimental design with defined input and output process variables is required to perform bioprocess modeling and optimization, as well as to better understand interactions between different bioprocess parameters [143]. The obtained bioprocess models are afterwards used for scaling up to a larger production scale, which is usually performed as seed train or a gradual increase in bioprocess production volume [144,145].

The usage of MCs in animal cell culture increases the surface–volume ratio of the cultivation vessel, which is of utmost importance for anchorage-dependent cells to provide sufficient area for their anchoring [72], providing a chance to obtain larger number of muscle cells in a lower production volume or through a lower number of seed train steps, resulting in saving and maximal exploitation of the expensive cultivation medium. The study by Bardouille et al. [146] that investigated myogenesis of murine myogenic cell line C2C12 and the secondary myogenic cell strains M12 (derived from a wild-type mouse) using the different types of MCs in the hollow fiber-spinner flask pointed out specific conditions during the first three hours of the bioprocess for cell attachment to the MC surface, including short agitation intervals at a low agitation rate followed by longer settlement phases. Moderate agitation during the seeding phase is required to maximize MC–cell interaction and homogeneity in cell distribution, while anchorage of the cells to MCs happens during the non-agitation periods [138]. The same authors concluded that reduction in the cultivation volume could also improve cell–MC interaction; hence, the recommendation is to reduce the medium volume in the seeding phase to half or a third of the production (working) volume. A similar protocol for cell seeding on MCs with intermittent stirring and resting intervals was applied by Confalonieri et al. [147]. The “adhesion program” with low-rate mixing cycles and six times longer rest cycles was applied by Rozwadowska et al. [148] to seed human satellite cells onto collagen-coated MCs. It is very important to optimize cell seeding conditions in order to provide maximal cell anchorage to MCs, since cell attachment efficiency significantly affects proliferation kinetics. Higher seeding efficiency at the beginning of the cultivation results in more rapid cell growth and higher cell concentration at the of the bioprocess [138]. In addition to the aforementioned seeding strategy with intermittent stirring, several other methods could be employed to increase seeding efficiency, including static seeding (without agitation) to maximize cell–MC contact time, as well as continuous agitation [137]. Depending on the cell line, it is important to optimize the cell seeding protocol in such a way to maximize cell–MC contact, but also to avoid MC aggregation [137]. Several other factors, aside from stirring strategy, also affect initial cell attachment on MCs during the seeding phase: MC material or coating, cell seeding density and initial concentration of MCs, the impeller shape and configuration, agitation speed and regime, as well as microenvironment conditions (medium composition, temperature, pH value) and medium volume [137,138]. Verbruggen et al. were the first to confirm the ability of bovine myoblasts proliferation on MCs in spinner flasks [140]. The results of this study have also confirmed bead-to-bead transfer of bovine myoblasts from one MC bead to another, which happens when cells bridge between the MCs or cells from one MC bead anchor to the other MC bead [149]. Bead-to-bead transfer indicated the possibility to add fresh MCs in order to obtain increased cell density in the lower production volume, hence overcoming the confluency limitations [137]. The increase of surface area per production volume, e.g., by adding fresh MCs, is one of the promising directions for proliferation scale-up when it comes to anchorage-dependent cells [72,150]. A significant increase in cell growth was observed when adding fresh MCs; hence, bovine myoblasts have shown similar behavior to hMSCs [72,149,150]. These results, although obtained in spinner flask conditions with limited control over temperature, O_2_ supply and nutrient availability and usage, could be used for further optimization and scale up of the proliferation process to a large-scale stirred-tank reactor (STR) [140].

The commercially available lab-scale bioreactor systems for TE and production of cell culture biopharmaceuticals include stirred-tank, hollow fiber, roller bottle, rocking-bed, fluidized-bed and fixed (packed) bed systems [151]. For example, different types of bioreactors have been employed for the expansion of hMSC—hollow-fiber, packed-bed, stirred-tank, rotating-wall vessel and vertical wheel bioreactors [137]. Bioreactor cultivation as a part of upstream processing usually includes three common modes of cell culture operation: batch, fed-batch and semi-continuous (draw-fill) mode. Lam et al. [152] have shown that the fed-batch feeding strategy could result in 70% less medium consumption compared to draw-fill operation mode, leading to significantly lower overall production costs, as well as reduced culture-related manipulation, which is especially important at the commercial scale. When it comes to CM production, fed-batch or continuous mode of operation would be appropriate considering the necessity to provide as uniform conditions as possible for cultivated cells, regarding both nutrient supply and waste metabolic byproducts removal [145,153]. Considering the adherent nature of the majority of relevant cell types needed in the production of CM, bioreactor systems suitable for CM production could be divided to static (with slow circulation of the cultivation medium through the cell layer in the form of packed-bed or 3D scaffold with attached cells) or dynamically mixed (stirred, shaken or fluidized) bioreactors [151]. A distinction should be made also regarding the bioreactor types that could be used in different phases of CM production, namely proliferation (expansion) and differentiation. Proliferation is aimed at obtaining as high cell density as possible; hence, the cultivation of animal cells on microcarriers in an STR, fluidized-bed reactor (FBR), rocking-bed reactor (RBR) or another type of dynamically mixed bioreactor would be appropriate for reaching the previously defined goal [29,144,154]. The static bioreactors with scaffolds or packed-bed structures for cell immobilization, with mild conditions in terms of shear stress due to slow medium circulation, support the differentiation of cultivated cells into myotubes, providing the possibility to mimic the actual conditions of myogenesis [145], especially when it comes to hollow-fiber reactors (HFR), where the role of blood vessels could be successfully mimicked by the presence of the hollow fibers for nutrient supply and metabolite removal [29,153]. However, considering the possibility to reach high animal cell density by applying the perfusion mode of operation in, e.g., packed-bed reactors (PBR) [145,153], this type of bioreactor could also find application in the proliferation of muscle cells or in the final step of the seed-train procedure, not only for obtaining 3D-cut meat constructs, as explained later.

### 3.2. Stirred-Tank Bioreactor

STRs provide a well-mixed environment with a possibility to precisely control bioprocess conditions, such as pH value, dissolved oxygen concentration and concentration of nutrients in the cell culture broth [138,145,155]. Although STRs provide mostly uniform conditions and a wide palette of possibilities when it comes to bioprocess monitoring and control, there are a few aspects that must be considered related to hydrodynamic conditions in the bioreactor. The presence of the inner moving parts of the bioreactor in direct contact with cells/MCs increases hydrodynamic stress for cells, especially at a higher agitation speed and increased fluid dynamic force, which could be detrimental for cell growth and later differentiation capability [156]. On the other hand, too low an agitation rate could result in cells and MC aggregation [151], as well as in poor mass transfer and consequently inhomogeneity in the cultivation medium, which could lead to different cell growth rates across the cell culture. One more reason for MC aggregation is confluency at the MC surface and possible multi-layered cell growth, resulting in the formation of larger MC–cell beads which are more prone to clumping [157].

Two strategies are usually applied to prevent MC aggregation: adjustment of the agitation rate and addition of fresh MCs to stimulate bead-to-bead transfer [149,158]. Although mixing of cultivation broth ensures uniform distribution of nutrients required by the cell culture, spatial distribution of shear stress caused by agitation is far from uniform, with localized “hot spots” near the stirrer and low shear stress zone near the top of the bioreactor vessel [159]. Mixing in the bioreactor containing suspension of MCs with adhered cells and non-anchored freely-floating cells needs to be intense enough to provide nutrients and waste metabolic products transfer between the cells and the surrounding medium [159]. However, intensive agitation can induce flow stresses responsible for deleterious effects such as cell death, reduction in cell growth and in some cases MC damage [77]. On the other hand, too slow agitation could induce excessive agglomeration of MCs and cells, leading to poor transfer of oxygen and other nutrients to cells, thus compromising cell viability [160]. One more thing that should be taken into account when optimizing cell culture exposure to shear stress through the optimization of stirring rate is also the possibility of unintended cell differentiation, which is undesirable in a bioreactor system designed to expand a homogeneous population of animal cells [161]. Silva Couto et al. [137] have identified several aspects to be considered when it comes to bioprocessing aimed at obtaining viable cells as the main products in STR: optimization of gassing strategy and agitation rate in order to define the minimal agitation level required, especially in MC-based culture, MC type, optimization of feeding or medium exchange regime, process robustness and reproducibility in order to ensure scalability without fundamental changes of cellular structure and properties. Breese and Admassu have performed modeling of the continuous STR for the proliferation of C2C12 mouse myoblasts on glass polymer-coated microcarriers in order to predict bioreactor performance [162]. The model was successfully validated, indicating the possibility to further develop the continuous bioprocess for cell harvesting and reinoculation to produce high density myoblast cell mass. STR was equipped with a marine impeller and ring sparger for air distribution. Rudolph and co-authors have cultivated murine cell line NIH-3T3 using Cytodex 1 MCs in a 5 L-STR [163].

Disposable or single-use options of STR are also commercially available and used in the production of biopharmaceuticals. The main advantages of disposable bioreactors include savings on utilities, labor and time related to cleaning and sterilization and significantly reduced contamination concerns due to pre-sterilized equipment and process monitoring sensors [164,165]. Furthermore, preparation time between batches is significantly shortened by applying single-use technologies instead of CIP (cleaning-in-place) and SIP (sterilization-in-place) procedures, making the process pipeline faster and more efficient and resulting in increased overall plant productivity [166]. Disposable parts of STR usually include system parts in direct contact with cultivated cells, such as vessel, probes and impellers. Hähnel and co-workers have evaluated several commercially available disposable STRs for mammalian cell cultivation [165]. A single-use, disposable STR vessel (Mobius CellReady 3 L, MilliporeSigma, Burlington, MA, USA) was recently applied for the expansion of bovine adipose-derived stem cells (bASCs) as precursors of fat and muscle tissue [167]. This study has given new perspectives on CM production by increasing the scale of a microcarrier-based (SoloHill Plastic microcarriers, Pall Corporation, New York, NY, USA) expansion bioprocess from 100 mL-spinner flask across 500 mL-spinner flask to 3 L-STR with a disposable vessel. Furthermore, the process intensification approach based on “surface + volume addition”, i.e., timely-defined addition of fresh MCs to support bead-to-bead transfer and medium exchange, has resulted in a significant fold increase of 114.19 ± 1.07 when it comes to bASC cell number [167].

### 3.3. Rocking-Bed Bioreactor

Vijay Singh was the first to describe the RBR or wave reactor intended for cultivation of animal cells [168]. The construction of the wave bioreactor consists of a flexible polymeric bag (usually made of polyethylene) placed on the rocking platform, with special ports allowing us to introduce air, oxygen or medium or to withdraw samples under sterile conditions. The chamber of the bioreactor is partially filled with medium and cultivated cells, while the rest of the space is filled with air which is being continuously passaged through the bioreactor headspace, in order to keep the bioreactor bag completely inflated [169]. Medium exchange through the bioreactor bag is also allowed; hence, different operation modes could be applied, including perfusion [170]. The contact surface between the medium and the air in the bioreactor bag provides oxygenation and gas exchange for pH control and CO_2_ removal, while the superficial contact between the gas and the liquid is being increased through the waves created by the rocking motion of the platform backward and forward, providing improved mass transfer. Rocking motion and creation of waves also improve movement of the cells from the bioreactor bottom through the medium bulk, thus minimizing mass transfer and temperature gradients [168]. In addition to good mass transfer characteristics, this type of bioreactor also provides mild shear-stress conditions for cultivated cells. Temperature control could be performed by placing the bioreactor system in the CO_2_ incubator, or by heating the rocking platform [168]. Bioreactor bags are usually disposable, facilitating bioreactor operation and mitigating necessity for sterilization, hence providing greater security and lower contamination risks [145]. High cell density cultivation of Chinese Hamster Ovary (CHO) cells was performed in the perfusion mode in disposable WAVE Bioreactor™ using “classical” tangential flow filtration (TFF) and alternating tangential flow system (ATF) consisting of the hollow fiber filter as the cell separation device [171,172].

### 3.4. Fluidized-Bed Bioreactor

FBRs are rarely applied in tissue culture, but with appropriate considerations, mostly regarding mass transfer, they could be successfully applied for the expansion of animal cell culture. FBR is characterized by the continuous upward medium flow through a layer of particles or immobilized cells allowing them to be suspended in a so-called fluidized layer [80]. In order to prevent cells washing out from the system due to upward medium flow, it is necessary to immobilize cells, either on the surface or entrapped within the particles made of different biocompatible materials [173], e.g., materials used for manufacturing of MCs or scaffolds. The advantages of FBR that qualify them as possible candidates for CM production include homogenous bed expansion behavior, related to good mass transfer characteristics, as well as lower shear stress compared to STR and simpler scale-up procedures [174]. This type of bioreactor is hence characterized by better mixing conditions due to constant flow circulation through the fluidized layer of particles, reducing emergence of temperature gradients due to the high heat transfer coefficient, as well as promoting homogeneous dispersion of the solid particles, together with better mass transfer in terms of nutrients and cell culture by-products, with simultaneous low hydrodynamic shear stress [173]. The absence of the inner moving parts used for agitation in FBR significantly simplifies their construction, lowers operational costs, and, as previously said, simplifies scale-up. Furthermore, FBRs represent well-mixed systems even without an internal mechanical agitation system, making it more suitable for cultivation of shear-sensitive mammalian cells [80].

The most important part of the FBR is the flow distributor, in charge of uniform distribution of cells/MCs and nutrients within the cultivation medium. Furthermore, consideration must be made regarding providing uniform mass/energy transfer, but also reducing turbulence and shear stress to a level amenable to the cultivated cells [151]. In order for the bioreactor to operate in the fluidized-bed mode, it is necessary to apply superficial velocity higher than the minimum fluidization velocity, in order to start bed expansion, but lower than the maximal fluidization velocity, which leads to cells/particles flushing out from the system. Hence, in order to maximize performance of the FBR, it is necessary to characterize the expansion properties of the fluidized layer (bed of particles), i.e., the relationship between the fluidized bed voidage (or bed height, as they are interrelated) and superficial velocity of the fluid flow [80].

Odeleye et al. [151] have investigated two types of flow distributor in the FBR: a vertically fluidizing distributor and a swirling flow distributor with 45° angled flow channels (enabling axial and radial mixing of the carriers, cells, nutrients and metabolites). Both flow distributors were constructed by additive manufacturing, with respect to sterilization requirements. The cultivation of MSCs in the designed FBR with custom-made and 3D-printed flow distributors resulted in increased cell attachment and proliferation on the commercially available MCs, opening the possibility to apply additive manufacturing to make bioreactor parts, or the whole bioreactor units, very quickly and relatively less expensively, which could be useful for investigation, modeling and optimization purposes when it comes to CM production at the lab scale. One of the options when it comes to maintaining cells in the fluidized state includes application of centrifugal force, e.g., in the continuous centrifugal bioreactors [175,176,177]. This type of bioreactor combines two effects to keep cells in the fluidized layer: drag force arising from the medium flow through the reactor chamber at flow rates necessary to sustain high-density cultures, which is balanced with centrifugal force, arising from the system rotation. In this bioreactor system, mass transfer between the cells responsible for nutrient delivery and by-product removal is shifted from diffusion to convection, hence overcoming the diffusional limitation of large cell constructs and eliminating the mass transfer limitations associated with other types of bioreactors for high-density animal cell culture [177].

Li et al. [178] have proposed a concept of a large-scale air-lift bioreactor for animal cell cultivation aimed at the production of CM, with the estimation that a single reactor with a volume of 300 m^3^ could supply the meat demand of 75,000 people. CFD (computational fluid dynamics) analysis was applied to optimize bioreactor design in terms of geometry and internal structure in order to provide optimal conditions considering mixing and hence mass transfer and energy dissipation rate. The bioreactor construction included microporous sintered ring spargers for air distribution in the form of sub-millimeter bubbles, perforated plates added to the riser for flow distribution and concentric conical flow diffusers in the separator (downcomer). Although this study was aimed at defining a bioreactor solution for the scale-up of CM production, the authors suggested revision of the proposed concept once new or updated information on animal muscle cell culture become available.

### 3.5. Perfusion Bioreactor

Development of biotechnological processes in general, with a special emphasis on animal cell culture, is aimed at defining bioprocess solutions that warrant high volumetric productivity, batch-to-batch consistency and homogenous product quality at low costs [179]. Walther et al. [180] have recognized two major needs for future biomanufacturing: increased flexibility and reduced cost of goods. Construction of a manufacturing facility still represents the main cost, requiring significant time and capital. The “village scale” concept was proposed by van der Weele and Tramper [181], where small-scale production facilities would be desirable from the technological and societal point of view when it comes to solving the global problem of CM supply. Although operational costs for some biotechnological products have decreased considerably, operating expenses remain high for many novel and non-standard products, such as CM. The shift from batch to continuous bioprocessing offers the possibility to minimize the costs of goods and maximize flexibility, with additional benefits including simplified scale-up, standardization and more consistent product quality. This transition has been successfully implemented in many branches of biotechnological production [180], such as in the production of therapeutic proteins [182]. A similar positive framework for continuous processing was given by Fisher et al. [183] for both upstream and downstream processes, with a potential to provide production flexibility, reduce product shortages and variability, simplify scale-up procedures, improve product quality, increase productivity, reduce facility footprints, and reduce overall production costs. However, the commercial implementation of continuous mode of operation or perfusion in mammalian cell culture, as well as in CM production, has not been simple and rapid compared to microbial fermentations due to much smaller processing capacity with much higher product value, more complicated quality standards and stringent regulatory requirements for animal cell culture products [184]. Mammalian animal cell culture can be more difficult to operate and maintain desired bioprocess parameters compared to microbial fermentations due to slower growth rates, complex media systems, and more demanding requirements when it comes to process parameter controls [185].

The bioprocess throughput, productivity and flexibility could also be improved by engaging perfusion in the seed train or production stage, implemented either in continuous cell culture [186] or concentrated fed-batch mode of operation [172,187,188] as a hybrid mode of perfusion and fed-batch production culture.

Perfusion culture as an alternative continuous processing approach [183] assumes the continuous removal of medium remains and extracellular metabolites from the bioreactor, with or without recirculation to maintain steady state conditions, including constant growth rate and cell density, as well as constant composition of the cultivation medium, provided by the continuous culture supplementation with fresh cultivation medium. Hence, the perfusion system should guarantee the main requirements of the continuous bioreactors: to supply cultured cells with fresh and nutrient-rich medium, to continuously remove spent medium and cell metabolites from the bioreactor and to maintain constant cell concentration in the bioreactor. One of the main parameters which should be maintained constant in a specified operational range is cell density, with cell removal if necessary (usually provided by so-called cell bleed), in order to avoid reaching critically high cell densities which could seriously limit bioprocess control [183] and which could conflict with the main requirement of continuous processing—to maintain similar conditions for each cell present in the perfusion culture. Xu et al. [184] have shown that perfusion culture can achieve higher CHO cell density, as well as higher volumetric and specific productivity in comparison with fed-batch bioreactor cultivation. A higher viable cell density and volumetric productivity of CHO cells can be achieved with long durations of up to months in continuous perfusion culture compared to fed-batch production cultures with durations of 10–20 days [185]. Some of the reasons for increased productivity when it comes to perfusion culture include the ability to remove waste metabolic products, providing healthier cell culture at higher cell densities, as well as the smaller facility footprint to obtain the same output as in the fed-batch production mode [186]. Perfusion culture provides more homogenous distribution of nutrients and waste metabolic products, as well as a higher nutrient consumption rate [187]. Konstantinov et al. [188] have identified several factors of decision whether to apply fed-batch of perfusion culture, including market size, existing and available infrastructure, historical experience and product stability.

Providing high cell density by different cell retention methods in different bioreactor types operated in the perfusion mode is considered a promising direction for the future development of CM production [28]. There are several possibilities for cell retention in the bioreactor, as a pre-requirement for engagement of the perfusion operation mode, which are usually based on filtration (using, e.g., alternating tangential flow (ATF) devices or cross-flow filters), centrifugation or gravity [172,189]. Generally speaking, Fisher et al. [183] have defined several requirements when it comes to cell retention devices at laboratory and commercial scale: ease of installation, maintenance and operation, reliability over long-term culture, permeability toward cell debris, macromolecules, waste and cell culture products, and non-adverse effect on cell growth.

There has been a recent interest in the application of perfusion cultures at the seed bioreactor (N-1) step [190]. In this way, “intensification” of the cell growth in this last seed step could be provided, with a possibility to achieve much higher final viable cell density in the seed train than the one that could be achieved using conventional N-1 seed culture methods. Hence, a much higher inoculation density of viable cells in the production bioreactor could be achieved compared to conventional inoculation densities. This contributes to the reduction in exponential growth phase duration and overall fed-batch process duration by 13–43% without affecting the final quality and viability of animal cells, thus improving the overall facility output [191,192]. Considering the significant influence of a seed-train procedure to the overall CM production duration [28], utilization of the perfusion mode of operation for reaching high cell density to provide a sufficient inoculum amount for the proliferation phase of CM production is one of the possible directions to investigate.

Perfusion or continuous mode of operation is also applicable for immobilized cell culture; hence, this mode of operation is being considered in combination with mechanical cell immobilization using packed-bed reactors (PBRs) and HFRs with the use of porous packing or membranes, respectively [177]. In vivo, cells are immobilized in tissues and organs and are perfused by lymph, blood, etc. In the in vitro conditions, immobilization is primarily used to increase the stability and process intensity of the culture, reducing the negative effects mostly arising from the shear stress to cells [193]. A continuous perfusion system in TE applications contributes to uniform distribution of nutrients in the tissue construct, preventing drastic changes in nutrient concentration, as well as accumulation of undesired growth or differentiation-inhibiting metabolic products [194]. Furthermore, fluid flow shear stress during perfusion results in mechanical stimulation, which could enhance cell differentiation [195,196], together or without the chemical differentiation stimuli [197,198] or additional mechanical stimuli [199]. Perfusion operation mode in combination with animal cell immobilization on edible scaffolds or MCs represent the most promising method of future CM bioprocessing, namely in the differentiation phase [144,145,153]. However, limitations of these bioreactors should be considered when it comes to CM production, mostly in terms of heterogeneous cell growth due to inactive portions of the biomass caused by membrane fouling (in case of membranes used for cell immobilization of medium transfer), mass transport limitations, and non-uniform nutrient and inhibitor gradients [177,200].

#### 3.5.1. Fixed-Bed (Packed-Bed) Bioreactor

PBRs with porous carriers or fibrous scaffolds provide high carrier/scaffold-specific area to volume ratio and support high density culture (up to 10^8^ cells/mL) in a fully controlled and automated environment, hence their application could be attractive for good manufacturing practice (GMP) facilities [201]. Fixed-bed reactors usually consist of two vessels: one packed or filled with porous matrix for cell immobilization, and the other conditioning vessel containing the culture medium, which are connected via the circulation loop. The oxygenated medium is pumped from the conditioning vessel to the packed-bed vessel upward through the fixed-bed layer and back, while the cells are retained in the packed-bed column [193]. The conditioning vessel could be operated in batch, fed-batch or the most commonly used perfusion mode. In the packed-bed vessel, axial flow of the medium through the center of the fixed-bed layer supplies the immobilized cells with nutrients and oxygen, providing simultaneous removal of waste metabolic products. For a larger scale of PBR, radial flow of the medium through the packed-bed layer should be provided due to possible oxygen limitations [193]. The medium flow rate should be precisely adjusted in order to avoid the emergence of axial or radial concentration gradients [202]. Furthermore, the two aforementioned vessels could be combined into one vessel in order to achieve easier operation, sterilization and scale up [193].

Porous solid carriers or scaffolds used for cell immobilization provide very large surface matrices and protect adherent cells against mechanical stresses [203]. While the cultivation of animal cells in suspension requires MCs with a diameter less than 0.3 mm, and FBRs operate with particles of diameter between 0.3 mm and 1 mm, particles with a diameter over 1 mm are suitable for application in packed-bed systems [193]. Overall scaffold/packed-bed dimensions should be in the range of several centimeters to decimeters when it comes to the production of structured CM constructs [204]. The matrix for fixed-bed is usually made of glass, cellulose, collagen, polypropylene, polyurethane or ceramics [193]; however, CM production should rely on edible matrices that could be retained in the final CM product [28,72]. Additional advantages of fixed-bed reactor systems include the possibility of reaching very high cell concentrations from low initial cell numbers, which significantly simplifies inoculum preparation and reduces the number of the required seed train steps. The large surface area for cell growth and expansion in PBR requires less frequent cell passaging, providing the possibility to reduce cultivation medium and operational costs [155]. The cultivation of animal cells on solid carriers in the packed-bed form implies reaching high cell density, which requires an intensive medium perfusion rate in order to keep cells’ viability since intensive bioreactor volume exchanges are needed to guarantee sufficient nutrient supply of cells and efficient removal of waste metabolites [203]. Mass transfer coefficients into macropores of the porous packing material, especially when it comes to volumetric oxygen transfer coefficient, are considered as the main limiting factor for reaching high cell density [205], with additional factors whose impact is less pronounced, including structure of the porous surface, packing density and bioreactor volume exchange rate [203]. The specific surface of the packed-bed, i.e., the area available for cell attachment, as well as the porous structure of the scaffold or MC bed, which should provide reaching high cell density and direct animal cell differentiation towards the final 3D CM construct, are among the most important parameters to be considered when applying fixed-bed bioreactors for the differentiation phase of CM production [28]. A PBR system with a polystyrene scaffold was successfully applied for the expansion of murine-derived MSCs, and the same study has shown a scale-up possibility of the PBR from 160 cm^2^ to 2800 cm^2^ of the useful cell attachment area [206].

Perfusion rate could be considered as one of the main parameters of the fixed-bed reactor cultivation, since it defines cell growth capability and hence the final product quality, with simultaneous strong impact on the overall bioprocess economics, considering the energy requirements necessary to provide sufficient perfusion rate, as well as the problem of effluent generation rate, affecting their amount and further recirculation or processing. Therefore, fixed-bed reactors at the industrial scale should operate with a perfusion rate as low as possible without compromising the quantity and the quality of the final product [179]. Furthermore, periodic perfusion flow was suggested to better approximate physiological conditions by setting the suitable fluidic environment [199].

#### 3.5.2. Hollow-Fiber Bioreactor

Hollow fibers represent semi-permeable membranes which allow the flow of the water and nutrients for cell growth, as well as the removal of metabolic products, while at the same time the membrane does not allow cells to pass through and can also act as a cell immobilization base [207]. The material used for manufacturing of hollow fibers could be customized, mostly in terms of pore size, to pre-determine which compounds or particles should be retained or pass through the semipermeable membrane [145]. The most commonly used materials are cellulose and polyethersulfone, which are hydrophilic and have a high-percentage porosity [169]. Knazek et al. [208] were the first to describe the HFR for animal cell culture, which proved a thesis that mammalian cells could attach to hollow fibers and grow in vitro. HFR could be organized in two different ways: (a) hollow fibers could be used for medium flow in order to provide cells that are placed on the outside of the hollow fibers, nutrients, oxygen and possibility to remove waste metabolic products; (b) cells could be inoculated inside the hollow fibers, while the medium circulates around the hollow fibers [209,210,211]. Considering that the first option has already been used to mimic blood vessels in TE [202] with nutrients, oxygen and metabolites exchange through the diffusion between the intra-capillary and extra-capillary spaces [145], CM production could have more benefits from this solution where the hollow fibers are used for nutrient delivery and removal of undesired metabolites. This system provides a cell density similar to the one in the solid tissues, as well as the similar physiological state of the cells regarding nutrient supply, waste removal and pH control [169]. Hence, the HFRs in the context of CM production are usually considered as the perfusion systems based on the principle of compartmentalization, where cells in high density are retained inside the bioreactor compartment, while the medium is perfused through the hollow fibers placed within the cell compartment, i.e., this system could be considered as intra-capillary inoculation with extra-capillary perfusion [144,145]. The HFR designed in the previously described way provides an almost shear-free environment for the proliferation of cells to near-tissue-like densities, with simultaneous simplicity of operation. Although this type of bioreactor could be successfully applied for the production of biopharmaceuticals, such as extracellular proteins, there are several disadvantages when applying them as the production system in which the cells/tissues are the desired product, which is the case in CM production. There has been spatial inhomogeneity when it comes to culture environment, especially in terms of the concentration gradient of nutrients and oxygen, as well as pH value [211]. That means that the cells placed nearest the surface of the hollow fiber experience optimal growth conditions, while moving away radially from the hollow fiber, concentration gradients decrease and their effects on cell growth become more pronounced [145]. The opposite situation is present in terms of waste metabolic products, whose diffusional removal is optimal near the hollow fiber, while their concentration gradient rises when moving away from the medium flow. Hence, problems with the monitoring and control of culture environment, mostly nutrient and by-product concentration, dissolved oxygen (DO) and pH value, represent the most important disadvantages of the HFR for the production of CM. Furthermore, scale-up of these systems is insufficiently investigated and problematic considering that, during the scale-up, the distance between the hollow fibers and empty space for cell growth have not been kept constant in most cases, hence the nutrient delivery and metabolites’ removal characteristics of the systems are significantly different at the larger production scale. The membrane structure of the hollow fibers is prone to fouling issues, which significantly reduces mass transfer ability and reduces the lifespan of the system. Hence, hollow fiber reactors with larger pore sizes have been developed recently in order to mitigate the membrane fouling problem by complete passage of the metabolic waste products, as the main sources of fouling, through the hollow fiber membrane, while still providing good cell retention characteristics [212].

Since vascularization of the 3D tissue constructs aimed at providing sufficient mass transfer of nutrients is of utmost importance, Bettahalli et al. [135] have developed poly(l-lactic acid) (PLLA) hollow fiber membranes for artificial vasculature. These hollow fibers were tested in the perfusion bioreactor during the cultivation of C2C12 muscle cells on the outer surface, while the medium flow was performed through the interior of the hollow fibers. Dynamic cell culturing has resulted in the successful transport of nutrients to the cells at the outer fiber surface, indicating the suitability of the produced hollow fibers for artificial vascularization of the tissue constructs.

Yamamoto et al. [194] have utilized magnetically labeled myoblasts (C2C12 muscle cells) to obtain multilayer tissue constructs formed around cellulose acetate hollow fibers, mimicking vascular tissue for medium supply, where the cell arrangement in the desired way was achieved using the magnetic force. The perfusion HFR system has provided a highly dense tissue construct, indicating the potential of the applied technology for the production of large-scale muscle tissue constructs.

Baba and Sankai [207] have shown the possibility to eliminate necessity for scaffolding during maturation and muscle tissue assembly by production and fusion of muscle cell spheroids (C2C12 muscle cells) in the perfusion system with hollow fibers for nutrients supply, vaguely resembling blood vessels. The additional advantage of the proposed system is the possibility to form the resulting tissue into any desirable shape. However, there is a necessity to consider mass and energy transfer problems, therefore the hollow fibers were placed in parallel at the same distance with a void which should be filled with cell spheroids of the same thickness to provide uniform mass transfer.

### 3.6. Scale-Up, Scale-Down and Scale-Out Options

The bioreactor scale could be classified as micro, benchtop/laboratory and pilot or industrial scale and it mostly depends on the application of the bioreactor. Scale increase or scale-up is usually required during the commercialization process, in order to provide sufficient amounts of the final product at the industrial scale for commercial use and placement on the market. On the other hand, scale-down is usually associated with a necessity to investigate bioreactor performance under different conditions by using representative small-scale bioreactor models and thus making R&D procedure significantly more cost-effective. Therefore, scaling down is usually aimed at modeling and optimization of the production process at the smaller scale, generating data useful for application on a large scale, or in case of investigating possible wider application of the certain bioreactor type [80]. The usual scale-up and scale-down approaches are based on maintaining the number of process/bioreactor parameters (design, geometry, hydrodynamic and kinetic parameters) similar across the different scales [213].

Rafiq et al. [214] were the first to report successful cultivation of hMSC on commercial plastic MCs in a 5 L-STR, making a successful scale-up in terms of cell viability and density compared to 100 mL spinner flasks. However, this study only monitored DO and pH value, without bioprocess control, leaving a significant space to further optimize bioprocess conditions in terms of stirring and gas exchange. Cunha et al. [215] managed to scale up expansion of MSCs on MCs from 0.12 L-spinner flask to 2 L-STRr. The scale-up criterion for stirring rate was selected according to Schirmaier et al. [216], using the minimum agitation required for full suspension of the MCs. Many studies have shown decreases in cellular yields during the production scale increase [217], mostly due to inappropriate choice of scale-up strategy resulting in inadequate suspension of cultured cells and/or MCs, e.g., scale-up strategy including the constant agitation speed, which results in the lower specific power insufficient for appropriate mass transfer in larger production volume [137]. Agitation speed should not be the only criterion considered when it comes to bioprocess scale-up since it could not be used to compare bioreactors with different geometry (different shapes of impellers and the ratio of their size to the bioreactor vessel dimensions) [137]. Specific energy dissipation rate or the specific power has also been suggested as a suitable scale-up criterion to be maintained constant during the scale-up procedure. However, good scale-up performance when using this criterion appears as a result only in the case of similar bioreactor geometry across different scales. Nienow et al. [218] have shown that N_JS_ (the minimum agitation speed for particle suspension) should be used as the primary scale-up criterion in bioprocesses based on MC culture of anchorage-dependent animal cells. It is expected to overcome the scale-up uncertainties and problems with a more profound understanding of animal cell culture dynamics, as well as with more precise bioprocess control [137]. Li et al. [178] have proposed a conceptual solution—large-scale air-lift bioreactor of 300 m^3^ for CM production sufficient for addressing nutritional needs of 75,000 people, with the following operational parameters considered as acceptable for animal cell cultivation: mixing time of 103 s, energy dissipation rate of 46 W/m^3^ and the mass transfer coefficient of 36 L/h.

Contrary to the proliferation scale-up aimed at increasing the production volume, the most likely future of the scaling up of the maturation phase of CM production is seen in scaling-out or parallelization [145], i.e., usage of a large number of small-scale units to obtain thicker-cut meat products of a mass up to several kilograms to ensure feasibility of further manipulation and processing [219]. Aside from avoiding the challenges of large-scale bioprocess development, this approach could result in more efficient delivery of the final product, lower capital expenses and a reduced environmental footprint. The usage of 3D scaffolds supports the formation of a muscle-like structure and contributes to the favorable textural and sensory traits of the final product [144,155]. On the other hand, significantly higher cell density in a muscle-like structure during maturation combined with static cultivation with immobilized cells significantly reduces mass and energy transfer. Hence, perfusion, or slow medium circulation through the cell multilayer, has emerged as one of the solutions. Although the construction of highly efficient perfusion bioreactors is a current hot topic in TE, the most important aspect is cost reduction of the overall maturation process to provide cost-effectiveness for its scaling out and acceptable price of the final CM product.

## 4. Bioprocess Conditions for Production of CM

### 4.1. Bioprocess Parameters

#### 4.1.1. Temperature

Optimal cell growth is inevitably linked to temperature as one of the key parameters responsible for the performance of metabolic processes. Although optimal temperature for mammalian cell culture should be routinely set to 37 °C in analogy with body temperature, the maintenance of this process parameter at the desired set point value is different for spinner flask bioprocesses usually carried out in incubators and for bioprocesses taking place in bioreactors [137]. In both cases, external temperature control is being performed, with the main difference in the medium used for heat exchange (gaseous medium consisting of air/CO_2_ mixture is used in incubators, while liquid medium (usually water) or heat exchange through contact of solid materials are used in bioreactor system). A temperature control system usually includes a temperature sensor responsible for real-time temperature monitoring and an automated heat exchange system, usually in the form of a heating blanket or water jacket, whose activation is being dictated by the signal from the temperature sensor and by the deviation from the temperature set point.

#### 4.1.2. pH Value

The pH value of the cultivation medium as an environment for cell growth and metabolism exhibits significant effects on biochemical and physiological processes in cell culture. In bioreactor cell culture, pH value should be continuously monitored using the pH sensor, while its control should be based on the deviation between the signal acquired by the pH sensor and defined set point depending on the cell line and cultivation conditions. STR and other types of dynamically mixed bioreactors with satisfactory mass transfer usually provide good pH control, with a certain problem regarding pH gradient arising from direct addition of acid, base or CO_2_ to the cultivation medium, which could result in localized cell damage. It is well known that the production of CO_2_, resulting in higher pCO_2_, and lactate due to cell respiration results in lowering of the cultivation medium pH value. Hence, it is recommended to optimize the bioprocess in order to minimize the accumulation of CO_2_ or lactate in order to avoid extensive addition of pH-regulating substances [220]. This becomes more difficult in large-scale and high-density cell cultures, since higher accumulation of pH-disturbing metabolites takes place in larger production volumes by the large number of cultivated cells, requiring the addition of large volumes of pH-regulating substances and potential osmolality problems due to accumulation of salts in the cultivation medium [221]. Additionally, gas stripping of CO_2_ from the cultivation medium becomes more challenging in the larger volume [222]. Bioreactor, sparger and agitator construction significantly affect a system’s ability to sparge CO_2_ from the cultivation medium; hence, it is recommended to carefully consider these configuration aspects during scale-up in order to avoid significant changes in pH value which could detrimentally affect cell growth and process productivity. If possible, it is also recommended to determine culture pH profile during cultivation at the smaller scale for the selected cell line and bioprocess conditions, with simultaneous process modeling under different conditions, in order to perform scale-up successfully [220]. While pCO_2_ is directly dependent on cell type and density, CO_2_ stripping ability depends on gas throughput and oxygen mass transfer coefficient. For example, the addition of antifoaming agents usually significantly reduces oxygen mass transfer rate, leading to an increase in air/O_2_ sparging rate to maintain the DO set point in the case of automatic DO control. The change in sparging rate results in different system’s ability of CO_2_ stripping, leading to pH value fluctuations which could be resolved by CO_2_ sparging [220]. The addition of sodium hydroxide is the most usual way to minimize CO_2_ accumulation due to the Henderson–Hasselbalch relationship [186].

Perfusion systems as continuous systems with exchange of the medium and necessity to establish steady-state conditions as soon as possible, on the other hand, require more precise pH control. The main problem with perfusion cultures is the accumulation of metabolic by-products; hence, their removal before medium recirculation would be desirable. Furthermore, online monitoring of glucose/lactate could be successfully applied for enhanced pH control, with a significant reduction in base addition [223,224]. Additionally, the control of glucose level in the cultivation medium directly affects lactate production rate and hence pH value; hence, bioprocesses with programmed addition of glucose in order to maintain the desired lactate concentration is another way to control pH value [220]. Xu and Chen have demonstrated that high-density cultivation of CHO cells could be performed with minimum or no pH control in fed-batch mode in STR (3 L and 200 L total volume) for 14 days, as well as in a continuous perfusion system for 40 days [220]. Culture pH value was maintained by controlling lactate and CO_2_ production by adjusting glucose feeding profiles, and an additional benefit was the lack of necessity to use online glucose/lactate monitoring systems. It was shown that consistency in cell metabolism results in pH value deviation in the narrow value range, reducing the necessity for external pH control and simplifying bioprocess modeling by eliminating the effects of pH-regulating substances’ type and quantity as independent variables to bioprocess responses. Different data regarding optimal pH value for cell types described in this study could be found in the literature, ranging from 6.7–7.8 [77,158,225]. High levels of ammonia and lactate as the main metabolic by-products present in the cultivation broth and affecting culture pH value are also identified as the main causes of increased osmolality with a negative effect on animal cell growth [137].

#### 4.1.3. Aeration and Agitation—Mass and Energy Transfer

Oxygen is one of the main nutrients in animal cell culture, necessary for metabolic processes in animal cells, an undisrupted course of energetic metabolism and aerobic respiration [226]. Wang et al. [227] have reported that oxygen in cell culture impacts morphology, growth kinetics, differentiation ability and metabolic profile of cultivated cells. Normoxic conditions for cell culture include dissolved oxygen (DO) concentration of 100% in cultivation broth in equilibrium, corresponding to the atmospheric conditions of 20–21% of oxygen present in the air. However, depending on their role and position in tissues, many cell types, e.g., stem cells, experience low oxygen concentrations designated as the hypoxic conditions or in situ normoxia [228]; hence, usually a lower concentration of DO (less than 100%) in the cultivation broth is applied during animal cell culture to better approximate the physiologic situation for a certain cell type [137]. Oxygen content and gradient or mass transfer coefficient is of utmost importance especially when it comes to in vitro tissue development [229], making it a highly relevant parameter to be thoroughly considered for the production of 3D CM tissue constructs. In addition to providing optimal concentration of DO in the cultivation medium, the important aspect is also the control of gaseous exchange during animal cell culture. Oxygen could be supplied in the form of pure gas or air in the headspace of the bioreactor system (space above the cultivation broth) or directly into the cultivation broth through sparging. When it comes to aeration, since overhead aeration usually cannot satisfy cells’ requirements for oxygen in high-density and large-scale animal cell culture, sparging intensity is of utmost importance to provide sufficient amounts of DO for cell growth and metabolism. Some of the authors have shown better performance in terms of growth kinetics at DO values of 100% [218], while the others have discovered that better results could be achieved at 25% DO [230]. The amount of DO in the cultivation medium is directly proportional to air sparging rate and agitation rate, as well as highly dependent on the cultivation temperature; hence, it is necessary to optimize the aforementioned cultivation conditions to achieve a satisfactory amount of oxygen in the cultivation medium, but on the other hand to prevent shear stress originating from intense sparging and agitation and the resulting cell damage. Additionally, an important aspect to consider is energy dissipation rate emerging from more intense sparging and agitation. In order to define sustainable bioprocess solutions for CM production, it is necessary to minimize energetic process requirements and hence optimize heating/cooling, aeration and agitation conditions, taking into account the principle of minimum energy consumption as well, besides the final product quality and quantity.

Mechanical stresses in the cell culture mostly arise from mixing (internal or external) and submerged aeration. When it comes to cell cultivation using MCs, these stresses include fluid (gas and liquid) motion relative to MCs and cells attached to it, contact between the MCs and impeller or between MCs themselves, as well as shear stress caused by bursting air bubbles [230]. The cell damage caused by the described mechanical stresses could happen while cells are still attached to the MC surface, or after detachment while the cells are freely suspended in the cultivation medium. When it comes to freely suspended cells, it is considered that the presence of the moving parts in the cultivation medium used for internal mixing, as well as small air bubbles, contribute to more pronounced shear stress for animal cells [231]. On the other hand, the culture of CHO cells has proven to be more resilient to shear stress caused by bursting small air bubbles when grown on MCs [232]. This indicates the possibility of applying microspargers for air distribution in the cultivation medium for MC-based animal cell culture in order to obtain smaller air bubbles, which not only provide more efficient oxygen mass transfer due to larger specific area, but also enhance cell density during cultivation on MCs by minimizing cell damage caused by hydrodynamic stresses [178]. Direct introduction of air in the cultivation medium is desirable not only from the point of view of providing sufficient amounts of oxygen, but also in terms of CO_2_ removal, since air stripping is the most convenient method for CO_2_ concentration control at a large scale [230].

Considering the cultivation of animal cells on MCs in an STR, there could be several time-scales observed associated with the operating protocols for specific bioprocess steps, such as seeding protocol and medium exchange protocol in the case of a semi-continuous mode of operation. Cell seeding onto MCs usually assumes several minutes of stirring followed by the longer settlement phase (usually 30 min) for the first period of bioreactor operation, typically 24 h [138,233]. Medium exchange in the semi-continuous animal cell culture requires the impeller to be stationary to allow the MCs to settle to the bottom of the bioreactor in order to perform medium exchange to minimize cell and MC loss. Both protocols involve multiple stoppings and restarts of the impeller, resulting in MCs and anchored cells lifting off the bottom of the bioreactor vessel and their inevitable movement towards the impeller blades and magnets, where the fluid forces are orders of magnitude higher than in the regions away from the impeller [234]. Hence, modeling of bioprocess mass and energy transfer when it comes to animal cell culture should be able to resolve the relevant time-scales of stress fluctuation over the duration of key protocol time-scales during the bioprocess course in the STR [159]. During animal cell cultivation using MCs, it is very important to maintain MCs suspended in culture medium with maximal stationary intervals not longer than 5 s to ensure uniform take up of nutrients and oxygen by cells. In order to provide the aforementioned conditions and keep MCs moving in suspension, as well as to provide each cell an opportunity for nutrient uptake, it is necessary to apply minimum agitator speed, usually designated as N_JS_ [235]. Ibrahim and Nienow [236] have compared N_JS_ as an operating bioreactor speed for the three-blade 45°-pitch segment impeller and a Rushton turbine impeller with six vertical blades. Although both impellers have shown the same N_JS_ value, application of the Rushton turbine impeller has resulted in a higher energy dissipation rate, which is in direct correlation with the potential for cell damage. Hence, it was concluded that a down-pumping pitch-blade impeller is a more suitable choice for cell culture. Heathman et al. [230] have shown that application of N_JS_ resulted in poor suspending of MCs in cultivation medium in 250 mL STR equipped with a three-blade 30° pitch down pumping impeller, followed by increased clumping, while the application of a doubled value of N_JS_ resulted in a significantly decreased growth rate. The value of 1.3 NJS has given satisfactory results in terms of cell growth, indicating that a moderate increase in agitator speed could enhance mass transfer while simultaneously maintaining cell viability and post-harvest quality when it comes to MC-based cell culture in STR. Berry et al. [159] have shown that the reduction in the impeller speed in the STR had decreased the overall shear stress within the cultivation broth, but it had increased the mean stress exposure of the MC-anchored cell population due to a redistribution of the MC beads at the bottom of the bioreactor, which represents an area of a high shear stress. However, the exposure of any single MC bead will probably be limited due to the shielding effect of the large numbers of MCs present in this region.

Another problem when it comes to ensuring high cell density is the generation of foam, mostly as a consequence of micro bubble presence in the cultivation medium and protein denaturation. Cell viability in the foam layer at the top of the cultivation medium is strongly endangered due to pH variations, direct exposure to gas phase or lack of nutrients in the foam layer [178]. The usual solution for foaming in biotechnological processes is addition of the antifoam agents as surface-active additives contributing to a decrease in surface tension and consequently foaming reduction. Their addition is usually controlled according to signals originating from the bioreactor foam level sensors. However, excessive amounts of the antifoam agent in the cultivation medium lead to air bubble coalescence and formation of larger bubbles with a smaller specific surface, resulting in reduced oxygen mass transfer, as well as an increase in shear stress for cultivated cells [178]. Furthermore, the excess of antifoam agents could also contribute to the formation of a thin layer on the cells’ surface, also resulting in reduced mass transfer. When it comes to MC-based cell culture, it has been shown that a smaller amount of antifoam agent could be used compared to cell culture without MCs. Additionally, spraying of the antifoam agent over the foam layer reduces its required amount to ppb levels, compared to direct addition of the antifoam agent to the cultivation medium where the required amount of the antifoam agent lays in the g/L range [178]. Considering the very high cost of the most usually used antifoam agent Pluronic^®^ F68, it is obvious that a reduction in its usage directly contributes positively to process economics when it comes to CM production, giving another reason to point the investigation of the muscle cell expansion phase in the direction of MC-based culture. Taking into account the deleterious consequences of foaming to process results in terms of cell density, the conclusion is that the application of the antifoam agent is necessary, with a requirement to minimize its addition to achieve satisfactory process cost-effectiveness. On the other hand, the aforementioned Pluronic^®^ F68 in small amounts is usually applied as a cell protectant in TE and cell culture. It contributes to a reduction in cell surface hydrophobicity and consequently lower affinity towards cell attachment to air bubbles. Hence, cell damage, assumed to originate mostly due to shear stress for air bubbles-attached cells from energy release during air bubbles bursting, is significantly reduced [230]. However, the addition of Pluronic^®^ F68 also reduces cell affinity for binding to MC surface, resulting in a higher number of freely-suspended cells, making cell culture more sensitive to shear stress. Hence, there is a necessity to optimize MC–cell binding conditions in the submerged culture with air sparging [230], since the air sparging will be necessary to scale up the production of CM.

The significant effect of process control on the reduction in batch-to-batch variation in terms of yield was confirmed by reducing yield variation among different cultivations under 15% [230]. Batch-to-batch variations mostly arise from the starting material (biocatalyst, i.e., cells and raw material) used for cultivation, as well as from inconsistent bioprocess conditions, resulting in non-homogenous product quality and traits. Hence, the role of precise bioprocess control is clear when it comes to the improvement of final product quality consistency. Other sources of batch-to-batch variations could be resolved by using specifically developed cell lines for CM production, while the omission of the serum from the cultivation medium, as the main source of batch variability, also significantly affects the consistency of long-term production processes, besides positive economic effect and reduced ethical concerns.

#### 4.1.4. Duration

Mammalian cell culture processes are usually characterized by a duration of 2–3 weeks of the bioreactor cultivation itself, with additional time required for inoculum preparation (seed culture propagation) and downstream processing [237]. Generally speaking, cultivation procedure, including both proliferation and differentiation phases in CM production, mostly depends on the cell type and origin, considering that cell doubling time significantly differs for the same cell type from different species [238], as well as for different cell types from one species [239]. Developing a bioprocess workflow for mammalian cell culture lasts at least 3–4 months, including at least 3–4 rounds of 10–12 bioreactor runs to be performed, considering the aforementioned 2–3-week duration of bioprocess in the bioreactor, plus 1–2 weeks for seed propagation [237]. A framework for minced CM production of one month for a production capacity of 20,000 L was given by van der Weele and Tramper [181], including upstream operations (cleaning, sterilization, filling, etc.) and cultivation itself. Specht et al. [28] have anticipated that CM production at the same production scale could take between 10 days and 6 weeks, including both proliferation and differentiation stage, mostly depending on the duration of the seed train procedure. Furthermore, cell doubling time is significantly dependent on growth conditions; therefore, the optimization of growth conditions should be performed to minimize cell doubling time and overall bioprocess duration aimed at defining cost-effective CM production process. Hence, a scale-down approach in terms of the development of microbioreactors which provide similar bioprocess conditions comparable to large-scale bioreactors could significantly reduce the time and cost necessary for bioprocess development, including multiple experiments under different conditions to acquire reliable and representative data necessary for bioprocess modeling and optimization [73,80,237].

### 4.2. Media

Generating exact and tissue-specific media is one of the biggest challenges when growing stem and progenitor cells in general. Specifically, in CM, considering not only the chemical and biological, but also the ethical aspects of the process, the use of some regular components of the cell media is brought into question [240,241]. Scientifically, stem cell upscaling requires basic water and nutrient sources such as lipids, glucose, and proteins to maintain them as healthy and functional—e.g., Dulbecco’s Modified Eagle Medium (DMEM/F12) supplemented with non-essential amino acids, low concentration of fibroblast growth factor-2 (FGF-2) and L-glutamine [34,36]. When it comes to CM, the highest struggle is differentiation yield increase and sustainability, which further requires media optimization. So, one must understand that basic stem cell science research differs from meat growing in a few aspects that will be addressed in this section.

Additionally, adult stem cell culture media can be supplemented with components such as different growth factors (GFs)—epidermal growth factor (EGF), transforming GF-β (TGF-β) and extra FGF-2, serum/serum replacer, heparin and extracellular matrix components [242,243] in order to improve the growth or behavior of cultured cells [36].

The complexity of the differentiation process is still to be studied, especially in terms of pathway inhibitors needed for myocytes, fibroblasts, adipocytes and blood cell-specific differentiation [244,245]. However, growth factors that are in use already, such as bone morphogenetic proteins (BMPs), epidermal growth factor (EGF), FGF and vascular endothelial growth factor (VEGF), insulin-like growth factor (IGF), platelet-derived growth factor (PDGF), and hepatocyte growth factor (HGF), are considered relevant and so far sufficient to encourage further investigation of small single molecules [246].

During muscle cell differentiation, myoblasts fuse into skeletal myofibers and this process requires few stages of supplementation. The first stage of differentiation into skeletal myotubes is initiated by placing satellite cells in a differentiation medium [38,247]. Satellite cells activation and proliferation are mediated by GFs such as HGF, TGF-β, IGF-1 and IGF-2, FGFs, and cytokines—tumor necrosis factor-α (TNF-α), leukemia inhibitory factor (LIF) [240,248,249,250] and different interleukins (IL) such as IL-6 and IL-1β, while IL-6, IL-4 and IL-15 influence myogenic differentiation as well [38]. FGF-2 is known as a powerful inducer of satellite cells’ proliferation and inhibitor of satellite cells’ differentiation in vitro [251]. IGFs stimulate satellite cells’ proliferation and differentiation [252] and protein synthesis [253]. TGF-β1 and myostatin are inhibitors of proliferation [254,255], TNF-α promotes myoblast differentiation [256], while LIF supports myoblast expansion, but not differentiation [240,249].

The cost of the proliferation and differentiation media, which differ in their supplements’ compositions, is very high considering the amounts and specificity of certain growth factors, pathway inhibitors, vitamins, hormones, amino acids given. It is estimated that from 55% to almost 95% of the products’ total cost accounts on cell media, while 95% of media costs refers to growth factors [142]. The final set up of the media needs to fulfill the requirements of separate processes during cell growth as well as quality of the final product. For example, an increase in glucose supplementation can cause cell acidosis and lower the quality of the final meat product [29], Vitamin D deficiency can cause severe degenerative problems in muscles [257], while Selenium and Vitamin E-insufficient supplementation can cause myopathies [258].

The first eight components medium (Essential media 8—E8) was a breakthrough discovery that lowered the costs and complexity of previously used media in cell culturing [34,259] and also opened a door to a fully controlled media processing. E8 is a serum-free, nutritionally rich media that contains three key signaling components: insulin or IGF1, FGF2, and TGF-β1 [260,261]. Recently, a novel medium formula (B8) was introduced, designed to support a high growth rate under low seeding density conditions [262] and was optimal for more than 100 passages of iPSCs. Additionally, research is being conducted on developing small molecule cocktails that can increase the yield and cellular survival during differentiation, such as the Rho Kinase inhibitors—ROCKi or a small molecule cocktail (chroman 1, emricasan, polyamines, and trans-ISRIB—CEPT) patents [263,264].

Fetal Bovine Serum (FBS) is an animal-based product rich in embryonic growth-promoting factors used in cell culturing as a main source of proteins, lipids, amino acids and other components that come along with the main nutrients [265]. Although the manufacturing processes of FBS are highly controlled, following sterile cellular biology of ISO standards, the chemical content of the FBS mixture is not fully controlled or measured [266]. In addition, using the supplement that directly causes cross-animal contamination in a final product violates the basic principles of non-animal meat production and presents a not fully controlled bioprocess [267]. Equally importantly, FBS is ethically not fully acceptable, since its manufacturing involves animal slaughter, which is one of the main aspects CM production aims to omit.

Thus, FBS is not appreciated, but still in use in CM, mainly because there are not sufficiently good alternatives available, even though there are companies actively working on defining new FBS alternatives [268,269,270]. Most of these solutions are proprietary. An important step forward to the more general use of FBS alternatives is performed through the partnership of Aleph Farms and Wacker, a protein tech supplier, aiming to build an open supply chain for serum proteins, i.e., the replacement of FBS [271].

Defining the total FBS compound composition would give a promising step of separating them into a chemically controlled mixture, which could then be used only in a cell-specific manner. The controlled manufacturing of FBS would also lower the cost of certain media and increase the yield in order to reach massive production.

There are a few other reasons for striving to eliminate FBS from the medium for CM production: the avoidance of possible contamination by transmissible agents, such as viruses, bacteria or endotoxins, a final product acceptable in a vegetarian/vegan diet, simplified downstream processing and less-burdened bioprocess effluents, which will contribute to bioprocess cost reduction, as well as a more cost-effective production process considering the cost of the cultivation medium at a large scale. Furthermore, the usage of serum is undesirable considering the sustainability of supply and batch-to-batch variation in composition, where animal-derived raw materials tend to be eliminated from the production processes.

The production of CM using bovine myoblasts currently requires 20% FBS and 10% HS (Horse Serum) in the advanced DMEM (Dulbecco′s Modified Eagle′s Medium) with the addition of antibiotics (usually 1% of penicillin, streptomycin, amphotericin) [272] in the proliferation stage. One of the ways to avoid the usage of serum in the cultivation medium is the development of serum-free adapted cell lineages, through the gradual adaptation of cells to serum-free conditions. The adaptation should be performed through a series of serum reduction steps, using the nutritionally rich growth medium to compensate for the serum absence [273]. Heathman et al. [230,274] have shown that advanced bioprocess control, especially in terms of DO concentration, had resulted in a significant hMSC yield increase in serum-free medium (PRIME-XV MSC Expansion SFM—serum-free medium) compared to FBS-supplemented medium. Kolkmann et al. [272] have investigated several commercially available media for the cultivation of bovine myoblasts in order to replace serum-amended DMEM. The results of this study have shown the potential of FBM (Fibroblast Basal Medium), FBM/DMEM and Essential 8^™^ Medium to become alternatives to serum-supplemented medium for bovine myoblast proliferation. However, further research and optimization are required, especially in terms of GFs necessary to achieve cell growth comparable to the one obtained using the serum-supplemented medium. Furthermore, the results of this study have revealed that the omission of antibiotics from the cultivation medium resulted in higher cell growth, indicating that antibiotics addition is not required for CM production using bovine myoblasts. Hence, a simplified downstream processing could be achieved, with a lower risk of development of antibiotic resistance due to exposure to antibiotic residues originating from CM products.

### 4.3. Bioprocess Monitoring

#### 4.3.1. Sensing Options

Monitoring the bioprocess of the CM production is one of the main steps that has to be followed in order to ensure production efficiency. That primarily refers to the use of sensors whose role is to define the most optimal production conditions that allow better process control, which further leads to a change in the cost of production itself [73].

Based on the way the sensors are connected to the bioprocess, they can be divided at in-line, at-line, or off-line. In-line sensors (also known as invasive, embedded, or in situ sensors) are in direct contact with the culture fluid, and they are located in the bioreactor interface. At-line sensors (also known as indirect sensors) require a special module for sample collecting and its analysis outside the bioreactor. If the measurement data of in-line and at-line sensors are recorded continuously and the response time of the sensor signal is small compared to the process dynamics, these two types of measurements can be considered as on-line. Every other type of measurement can be observed as off-line [274,275].

#### 4.3.2. Sensors for Temperature, pH, DO, CO_2_ and Biomass

Bioprocess parameters that play a crucial role during cell cultivation in bioreactors include several physical and chemical variables such as pressure, temperature, pH, the concentration of DO and CO_2_ as well as amount and composition of nutrients in the cell culture medium [276,277]. Monitoring of these mentioned parameters using sensors significantly increases the understanding of the entire bioprocess, which further permits its optimization and productivity.

Cultivation temperature is essential to ensure optimal cell growth rate and viability. It primarily depends on the types of cells used for cultivation, and for all mammalian cells, it is about 37 °C [278]. Precise control of cell culture is very important, given the fact that even small changes in its range can negatively affect the optimal growth of cell cultures. For example, Weidemann et al. showed that temperatures below 37 °C for cultures negatively affect cell growth, as well as glucose utilization [279]. On the other hand, in the paper given by Furukawa and Ohsuye, it has been shown that temperatures above 37 °C can cause a large loss in cell vitality and cell production [280]. Due to all of the above, it can be concluded that the high precision and accuracy of temperature sensors are of great importance to avoid losses of cellular viability [279].

There are two main types of temperature probe usually used for bioreactors: (1.) resistance temperature devices (RTDs) and (2.) thermocouples. RTDs are based on the measurements of the change in electrical resistance in metal (Pt, Zn, Ni, or Cu wire), which is known to change with temperature. These sensors are known as highly accurate and stable with fast responses, which is the reason for their wide usage in bioreactors. On the other hand, thermocouples are the most widely used sensors for monitoring temperature. Those devices are constructed of two different electrical conductors, and they are characterized by a relatively low price, which makes them affordable. Nevertheless, their sensitivity is significantly lower than in RTDs [281].

One of the basic variables in the culture medium is pH, as described in detail in Section 4.1.2, and as such it needs to be constantly monitored by sensors [282] and optimized as needed, usually by using a previously formulated buffer system. This type of control is necessary since, in general, all biological processes are extremely sensitive to acid–base chemistry. When it comes to cell culture, pH can deliver information about the rate of cell growth during cultivation along with their metabolic processes (reduced values of pH can indicate the accumulation of waste products (with acidic properties) like for instance carbonic acid, lactates, etc.) [283]. Medium for cell cultivation is known to be rich with amino acids, vitamins, salts (and other molecules needed for the cells), as well as an inorganic buffer component—NaHCO_3_. NaHCO_3_ strongly depends on the content of CO_2_, the constant supply of which during cultivation is necessary for pH homeostasis. There are two most common types of pH monitoring sensors used in bioreactors: 1. optical and 2. electrochemical. Optical pH sensors are based on measuring the optical performances or detection of the fluorescence of the indicator color located on the sensor surface. In contrast, electrochemical sensors consist of ion-selective working electrodes (silver or silver-chloride) that measure the potential change between the internal solution and the analyte across the membrane compared to the potential of the reference electrode [71,278].

Alongside pH, dissolved oxygen (DO) is also an excellent indicator of cell growth. Oxygen is a molecule necessary for the respiration of mammalian cells, and its concentration in the medium decreases with the increase in biomass. Additionally, the respiration of cells leads to the release of lactic acid as a product of cellular metabolism, which further leads to a decrease in the pH of the cellular medium. Furthermore, both DO and pH levels may provide information on cell entry into the apoptosis process, as previous research has shown a link between acidification and cell death [284]. It is important to note that different cell lines usually used during cultivation can have different rates of oxygen usage, and their needs for oxygen are different. All of the above testifies to the importance of controlling and monitoring dissolved oxygen during cell culture. Three types of sensors are most commonly used to measure DO in bioreactors: (1.) optical, (2.) electrochemical, and (3.) paramagnetic. DO measurement with optical sensors is based on quenching the photoluminescence of an oxygen-sensitive indicator. These sensors are well known for their long shelf life compared to the other two types used in bioreactors, but their response time is somewhat slower [275]. In contrast to optical sensors, electrochemical sensors for monitoring the concentration of dissolved oxygen have a short shelf life. These sensors are known to have a short response time and good compactness. Most of these sensors can be found in bioreactors constructed of the anode (zinc or lead) and cathode (golden or silver) positioned in the electrolyte solution. Two types of metals are used to fabricate the cathode and anode in order to have a different reaction with the electrolyte, which further leads to the appearance of an electromotive voltage that is proportionate to the DO [285]. The principle of sensors based on paramagnetism is based on the fact that O_2_ is a paramagnetic gas present in the bioreactor, which allows O_2_ to be attracted to a strong magnetic field [71,285].

Dissolved carbon dioxide (CO_2_) is also one of the critical parameters in the production processes of cell cultivation. Since it highly affects other process parameters (i.e., pH), this molecule greatly influences the various metabolic pathways involved in cell growth. It is known that high concentrations of this molecule initiate the inhibition of mammalian cell growth [286]. Despite the need for strict control of this process parameter, its determination is not that easy due to its high reactivity with water and cultivation media containing NaHCO buffer systems [287]. The most commonly used sensors in the bioreactors for monitoring dissolved CO_2_ rely on the electrochemical approach. Such sensors utilize an optical system for indirect measurements of partial pressure of CO_2_, which relies on changes in pH in bicarbonate solution [288]. In addition to the electrochemical ones, it is possible to use optical sensors for CO_2_. There are a number of limitations associated with this type of sensor, so their use in bioreactors is not that popular. One significant limitation of optical CO_2_ sensors relies on a relatively slow response time—caused by the slow diffusion of CO_2_ molecules throughout the selective membrane. This selective membrane is also known to have a quite short shelf-life, so it needs to be changed from time to time, which further requires the installation of new membranes and recalibration of the sensor. Another issue with optical CO_2_ sensors is that they are unstable at low temperatures [289].

Since biomass represents the growth of cells in the bioreactor during their cultivation, this parameter is considered to be one of the most important ones to monitor during cultivation time. In addition to the cell concentration, it is also important to monitor their viability in the bioreactor. There are different approaches to biomass assessment, involving the use of different monitoring sensors [274]. Manual cell counting, near-infrared (NIR) spectroscopy [290] and dielectric spectrometry [291] are some of the direct methods, while methods based on measuring gasses released during bioprocess [275,292], glucose uptake [293] and redox potential measurements [294] are indirect methods that can be applied. Integrating these techniques remains a challenge.

Measuring the viability of a cell culture system can be achieved by measuring the linear relationship between the optical signal and the biomass concentration, with the biomass assessment itself being determined based on biomass and lactic acid production [295,296]. Additionally, multifunctional platforms for measuring biomass, pH and O_2_ are used for biomass measurement [297].

As noted in Djisalov et al. [73], impedimetric sensors have great potential for measuring biomass. In addition to the principle based on radio frequency where the cell number directly affects the dielectric permittivity and the capacity of the measured impedance, the possibility of estimating biomass in cell suspension, i.e., in cultivation medium with cells seeded on MCs makes this method very applicable.

#### 4.3.3. Commercially Available Sensors

According to recently published reviews on the topic [73,285], commercially available temperature sensors can be divided into two categories based on the principles of resistance and thermocouple [298]. Resistance sensors include platinum, nickel, TSic and United Electric Controls [298,299]. Thermocouple sensors cover IST, Rosemount^TM^**_,_** Krohne, Pyroscience and Burns Engineering [298,299,300,301,302]. All the above-mentioned sensors have different accuracy and operating ranges.

Based on optical and electrochemical principles, commercially available pH sensors differ in design as well as in measuring ranges and response times. Within optical sensors in Pyroscience, the following are available: pH Sensor Spots, pH Flow Through Cell, pH Sensor Cap for Underwater devices [301]. PreSens sensors include pH-1 SMA LG1, Self-adhesive pH Sensor Spots SP-LG1-SA, Single-Use pH Flow-Through Cell FTC-SU-HP5-S, and Profiling pH Microsensor PM-HP5 [303]. Sensors based on the electrochemical principles include pH Probes and Bioreactor pH Probe [304,305].

Within DO sensors, optical oxygen sensors are mostly used due to their cost, lifespan and accuracy. Known brands are Mettler Toledo and PreSens Oxygen Sensors [306]. Besides optical oxygen sensors there are sensors based on Paramagnetic Cells Technology and Electrochemical sensors [307,308].

Commercially available CO_2_ sensors consist of Optical and Potentiometric sensors. Among optical PreSens CO_2_ Sensors are widely used [303,309]. These sensors are characterized by CO_2_ sensors with solid electrolyte and have a short response time for the in situ measurements. Additionally, these sensors have been miniaturized and the selectivity and sensitivity of IR sensors have been improved.

The sensors for these parameters have been developed in the direction of the integration of two or more sensors within one module. Easy and simple mounting or integration of such sensors within the bioreactor is one of the goals of the development of sensors for CM cultivation [73,285].

### 4.4. Bioprocess Kinetics

The characteristic animal cell culture growth curve is defined according to the sigmoidal pattern [310]. The first growth phase is associated with the cell adaptation to the environmental conditions and depends on the nutrient availability that supports the next phase, proliferation. The time required for the cell adaptation varies depending on the initial cell density and the subculturing regimen. The logarithmic phase is the most intensive growth stage characterized by an exponential increase in the cell concentration. Cell proliferation significantly decreases in the stationary growth phase when the number of cells dividing and dying becomes equal. The stationary phase ends with the decline stage [310]. Kinetic analysis of the cell cultivation technology includes calculations and defining mathematical relations between the key bioprocess factors, cell growth, nutrient consumption and metabolite accumulation. On the other hand, the greatest challenges in defining the bioprocess models are their complexity and lack of adequate measurement methods for the key bioprocess factors, since expansion is influenced by multiple parameters and simplified mathematical models cannot be applied [311]. The most suitable modeling approach describing the animal cell culture is kinetic modeling based on the determination of parameters characterized by dynamic change during the cultivation, including cell growth, cell death, and metabolite production/consumption [312]. Although the kinetic models could include metabolic reactions, the main differences from metabolic flux-based models take into account enzyme kinetics and consist of complex non-linear equations. Different kinetic models could be found in the literature, separated into several groups: structured or unstructured (considering the intracellular level) and segregated or unsegregated (considering the heterogeneity in cell population). The most common method available in literature data is the unstructured–unsegregated approach based on Monod-type equations describing the relation between the cell growth and substrate availability. In the category of unstructured–segregated models, one of the promising solutions is based on Monod growth kinetics considering multiple cell populations in different cell cycle phases [313]. On the other hand, structured–unsegregated models take into account specific intracellular processes by observing cell populations as homogeneous. A similar methodology is used for structured–segregated model definition with the exception of taking into consideration different cell populations [312]. Sidoli et al. [314] generated a complex model including more than 700 factors, based on a population balance model and a detailed single-cell model. One of the kinetic models developed by Galvanauskas et al. [311] included describing the nature of dependency between the aggregate growth and cell proliferation that enables the predictions in terms of aggregate size. This kind of approach is of great importance for cell concentration maximization in the expansion phase by using mathematical relations and defining optimal aggregate break-up and glucose and glutamine feeding strategies [311]. The multi-omics data integration and overall multiscale modeling in mammalian cell cultivation technology are defined as powerful tools for improved parametrization and predictive kinetic model generation. The innovative approach considers and integrates several key bioprocess aspects regarding signaling, regulatory actions and product characteristic relaying on intracellular interactions and cellular metabolism [312]. Furthermore, computational modeling could be applied to assess the effects of a range of parameters to cell growth, proliferation and differentiation, as well as tissue formation, with simultaneous savings of resources and significantly decreased R&D costs. Results in this field aimed at the advancement of CM production should be delivered by the Cultivated Meat Modeling Consortium (CMMC—https://thecmmc.org/, date of access 20 January 2022) and several other research groups.

### 4.5. Bioprocess Effluents

One of the highlights and possible main advantages of CM production over conventional methods includes a reduction in greenhouse gases emission [29]. On the other hand, the main challenges of the alternative way for CM production are high energy demands. Significant energy supply is described as a key factor for maintaining the controlled production conditions necessary to replace the animals’ biological functions [315]. The main bioprocess effluents from CM production include heat and metabolites, with the emphasis on CO_2_ as a result of energy generation [20,316]. On the other hand, accumulation of metabolic byproducts, lactic acid and ammonia could lead to cell inhibition [144]. The possible waste management approach considers the disposal or upgrading of generated effluents by valorization in some other production processes. For instance, lactic acid is a product of industrial interest with multiple applications. Recent trends in lactic acid production focus on defining eco-friendly processing methods, relying on the conversion of waste materials and giving advantage to the fermentative over the chemical approach [317]. Valorization of lactic acid as an effluent in CM production could be a significant contribution to the green production strategy. The same waste management principles, with the additional possibility of recycling media, could be considered when it comes to the unutilized nutrients, including glucose, amino acids, carbohydrates and proteins [144,318], and generated metabolites such as ammonia. Haraguchi et al. [69,319] have proposed an interesting in vitro “symbiotic recycling system” composed of mammalian cells and algae, resulting in significantly reduced glucose consumption by the C2C12 cells and rat cardiac cells and therefore lower metabolic production of lactate and ammonia, with simultaneous generation of a larger thickness tissue [69]. The downstream process should be created to enable the recycling of the valuable components and removal of the unwanted ones. Potential processes to be considered include membrane filtration, (electro)dialysis, precipitation, solvent extraction, and adsorption [144]. The development of sustainable CM production implies minimization of carbon footprint and effluents by creating a viable bioprocess solution integrated in the concept of circular economy [20,316]. The biotechnological production design addressing environmental stability and economic viability is imperative from the aspect of CM production scale up and reaching an industrial level.

**Figure 2:**
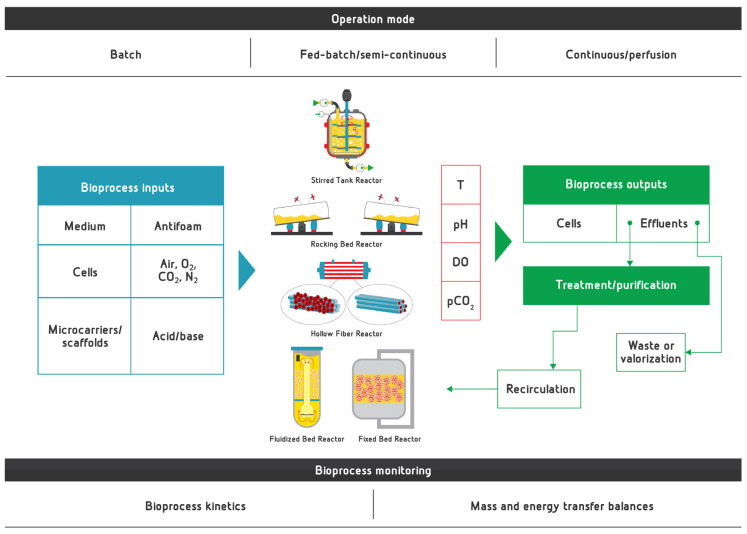
General considerations for bioprocess aimed at cultivated meat production.

Figure 2 shows the bioreactor types described in Section 3 and summarizes the various aspects discussed in detail in Section 4.

## 5. Bioengineering Aspects to Be Considered in Future Development of CM Industry

On a global scale, the CM industry is still at the proof-of-concept stage, despite the individual breakthroughs by several companies such as Aleph Farms, Mosa meat, Eat Just and others. There are some lines of thought that claim that CM will most likely never achieve a status of a cost-competitive food [320,321]. Indeed, there are still many challenges that the nascent CM industry would need to overcome, primarily associated with the metabolic inefficiency, shear-induced cell damage and low growth rate that limit the up-scaling of the bioreactors to be used. One way out of this problem may be to follow the scaling-out approach or parallelization we describe in the review.

Further improving the proliferative capacity of the used cells and creating immortalized cell lines of different livestock species is another research direction that needs to be explored intensively.

In order to reduce the costs of the cultivation media and accommodate to the slaughter-free criteria the whole CM field follows, it is necessary to intensify research on the FBS alternatives and replacement of animal-derived amino acids and protein growth factors (GF) with plant protein hydrolysates. It is worth considering the use of machine learning and artificial intelligence in the development of such hydrolysates and for the design of recombinant proteins that may be used as GF replacements, as well as for other aspects of CM bioprocess workflow development [322].

When aiming to reduce the overall costs related to CM bioprocess, it may be advisable to search for methods to reduce the CM production duration, such as utilization of the perfusion mode of operation for reaching high cell density to provide a sufficient inoculum amount for the proliferation phase of CM production. The use of perfusion operation mode in combination with animal cell immobilization on edible scaffolds is most likely the most promising way of future CM bioprocessing, but it still needs further improvements, mostly in terms of heterogeneous cell growth due to inactive portions of the biomass caused by membrane fouling, mass transport limitations, and non-uniform nutrient and inhibitor gradients.

One approach worth following is also to improve the bioprocess monitoring options, using advanced online sensing features. Currently the best options for developing advanced versions of different sensors are the scale-down approach and mathematical and CFD models where the model estimates are rigorously benchmarked to the empirical data obtained in microfluidic bioreactors, i.e., microbioreactors. Better sensors for nutrients and metabolites, such as ammonia, may allow for the cultivation medium recycling where the sensors (in combination with filtering) can be used to evaluate the levels of metabolic waste products (which need to be removed), non-metabolized components such as salts, buffers, and GFs (which can be retained and recycled), and the depleted nutrients such as sugars and amino acids (which can be replenished as they are consumed by the cells).

Mathematical modeling is of high importance in further improvement of the CM bioprocess kinetics. Significant efforts should be invested into devising precise computational models that assess the effects of a range of parameters on cell growth, proliferation and differentiation, as well as tissue formation, which could further lead to the savings of resources and decreased overall costs.

In summary, associated costs of the CM bioprocess are currently still extremely high, even if one consults the most optimistic techno-economic analyses [323]. This may lead to difficulties for the CM companies to deliver on the return of investment expected by their venture capital (VC) backers. This may further lead to a significant reduction in VC motivation for further investments. Therefore, it is necessary to have research pipelines that are not directly associated with private funding and that can allow for the whole scientific community to contribute to developments in the CM field, one of the most exciting but admittedly also one of the most ambitious research fields ever to exist.

## Data Availability

Data sharing not applicable. No new data were created or analyzed in this study.

## Abbreviations

ATF	alternating tangential flow system
BAMs	bio-artificial muscles
bASCs	adipose-derived stem cells
BMPs	morphogenetic proteins
CA	cellular agriculture
CFD	computational fluid dynamics
CHO	chinese hamster ovary cells
CM	cultured meat, cultivated meat
CMMC	cultivated meat modeling consortium
CIP	cleaning-in-place
CXCR4	C-X-C chemokine receptor type 4
DMEM	Dulbecco’s Modified Eagle Medium
DO	dissolved oxygen
ECM	extracellular matrix
EGF	epidermal growth factor
ESCs	embryonic stem cells
FACS	fluorescence-activated cell sorting
FAPs	fibro-adipogenic progenitors
FBM	fibroblast basal medium
FBR	fluidized-bed reactor
FBS	fetal bovine serum
FGF	fibroblast growth factor
FGF-2	fibroblast growth factor-2
GFs	growth factors
GMP	good manufacturing practice
HA	hyaluronic acid
HFR	hollow-fiber reactor
HGF	hepatocyte growth factor
hMSC	human mesenchymal stem cells
IGF	insulin-like growth factor
iPSC	induced pluripotent stem cells
LIF	leukemia inhibitory factor
LOC	Lab-on-a-Chip
MAPK	mitogen-activated protein kinase
MCs	microcarriers
MOs	microorganisms
MRF	myogenic regulatory factor
MSCs	mesenchymal stem cells
MyoD	myoblast determination protein 1
PBR	packed-bed reactor
PDGF	platelet-derived growth factor
PDMS	polydimethylsiloxane
PEG	polyethylene glycol
PGA	polyglycolic acid
PLA	polylactic acid
PNIPAM-MCs	poly(*N*-isopropylacrylamide) grafted MCs
R&D	research and development
RBR	rocking-bed reactor
RTDs	resistance temperature devices
SCNT ES	somatic cell nuclear transfer embryonic stem cell
SDG	sustainable development goal
SIP	sterilization-in-place
STR	stirred-tank reactor
TE	tissue engineering
TFF	tangential flow filtration
TGF-β	transforming GF-β
TNF-α	tumor necrosis factor-α
VEGF	vascular endothelial growth factor
VC	venture capital
